# Rab-GTPase binding effector protein 2 (RABEP2) is a primed substrate for Glycogen Synthase kinase-3 (GSK3)

**DOI:** 10.1038/s41598-017-17087-6

**Published:** 2017-12-15

**Authors:** Lisa Logie, Lidy Van Aalten, Axel Knebel, Thomas Force, C. James Hastie, Hilary MacLauchlan, David G. Campbell, Robert Gourlay, Alan Prescott, Jane Davidson, Will Fuller, Calum Sutherland

**Affiliations:** 1 Division of Molecular and Clinical Medicine, University of Dundee, Dundee, UK; 2 MRC Protein Phosphorylation and Ubiquitylation Unit, University of Dundee, Dundee, UK; 3 Department of Cardiovascular Medicine, Vanderbilt University, Tennessee, TN USA; 4 Division of Signal Transduction and Therapy, University of Dundee, Dundee, UK; 5 College of Life Sciences, University of Dundee, Dundee, UK; 6 Division of Cancer, University of Dundee, Dundee, UK

## Abstract

Glycogen synthase kinase-3 (GSK3) regulates many physiological processes through phosphorylation of a diverse array of substrates. Inhibitors of GSK3 have been generated as potential therapies in several diseases, however the vital role GSK3 plays in cell biology makes the clinical use of GSK3 inhibitors potentially problematic. A clearer understanding of true physiological and pathophysiological substrates of GSK3 should provide opportunities for more selective, disease specific, manipulation of GSK3. To identify kinetically favourable substrates we performed a GSK3 substrate screen in heart tissue. Rab-GTPase binding effector protein 2 (RABEP2) was identified as a novel GSK3 substrate and GSK3 phosphorylation of RABEP2 at Ser200 was enhanced by prior phosphorylation at Ser204, fitting the known consensus sequence for GSK3 substrates. Both residues are phosphorylated in cells while only Ser200 phosphorylation is reduced following inhibition of GSK3. RABEP2 function was originally identified as a Rab5 binding protein. We did not observe co-localisation of RABEP2 and Rab5 in cells, while ectopic expression of RABEP2 had no effect on endosomal recycling. The work presented identifies RABEP2 as a novel primed substrate of GSK3, and thus a potential biomarker for GSK3 activity, but understanding how phosphorylation regulates RABEP2 function requires more information on physiological roles of RABEP2.

## Introduction

Glycogen synthase kinase-3 (GSK3) was originally identified as a regulator of glycogen synthesis but is now known to influence many important cellular processes^1–3^. The two mammalian GSK3 genes (GSK3α and GSK3β) are >90% identical in their catalytic domain sequences. GSK3β deletion results in postnatal lethality, with multiple developmental defects and loss of hepatic function^4^. In contrast GSK3α null mice are viable and relatively healthy with some defects in glucose metabolism^5^. However, the GSK3α null mice have a shorter lifespan and are more prone to chronic age-related diseases^6^. This implies that the GSK3α and GSK3β isoforms contribute to differing aspects of healthy ageing in mice. Interestingly alterations in GSK3 activity are found in age-related human diseases including diabetes, cancer, Alzheimer’s disease (AD), schizophrenia, Bipolar Disorder, inflammation, and cardiac hypertrophy^3,7–9^. Partial deletion (pharmacological or genetic) of GSK3 reduces the development and/or severity of models of these diseases^3,10–12^ indicating a key contribution to their initiation/early progression^3^. Several major pharmaceutical companies have developed selective and potent GSK3 inhibitor small molecules. However the wide spectrum of physiologically important GSK3 substrates combined with the lethality of GSK3β gene deletion^4^ indicates that this enzyme is vital for many biological actions, and seriously dampens enthusiasm for use of global GSK3 inhibition in the clinic. That said, GSK3 inhibition was found to be relatively well tolerated in Phase I human trials aimed to establish dose tolerance for use as an adjunct to platinum-based therapies in cancer treatment^13^. However, a more disease selective intervention would be more elegant, less likely to have toxicity issues and have clear clinical potential. At present there is little data on disease specific substrates of GSK3, the exception possibly being the Alzheimer’s disease tangle protein, tau^14^.

GSK3 is an unusual kinase in that the majority of its targets require prior phosphorylation by an alternative kinase to generate a GSK3 consensus sequence (Ser/Thr-X_3or4_-PhosphoSer/Thr, X is any amino acid)^15^. This is termed priming and enhances phosphorylation of peptide substrates of GSK3 by more than 1000-fold. Different groups of GSK3 substrates have distinct ‘priming kinases’, and this regulatory mechanism provides opportunities for physiological, pathophysiological or pharmacological manipulation of specific substrates primed by a common protein kinase, independent of direct GSK3 regulation. It remains to be seen whether there are disease related priming defects that enhance specific subsets of GSK3 substrate phosphorylation.

In summary establishing which substrates of GSK3 mediate the pathophysiological actions of this enzyme, and identifying the regulatory details of these substrate phosphorylations, could provide novel disease specific therapeutic options. In this paper we identify a novel GSK3 substrate, RABEP2 (a proposed regulator of Rab signaling), map the residues targeted by GSK3 in cells, and show priming is required for GSK3 to regulate RABEP2 *in vivo*. This is a relatively poorly studied protein with the potential to regulate key membrane associated biological processes. Screening of RABEP2 phosphorylation in GSK3-associated diseases should help establish whether the regulation of RABEP2 is altered in human diseases where GSK3 activity (or specific priming events) is increased.

## Results

### Identification of a novel GSK3 substrate using KESTREL

**Figure 1:**
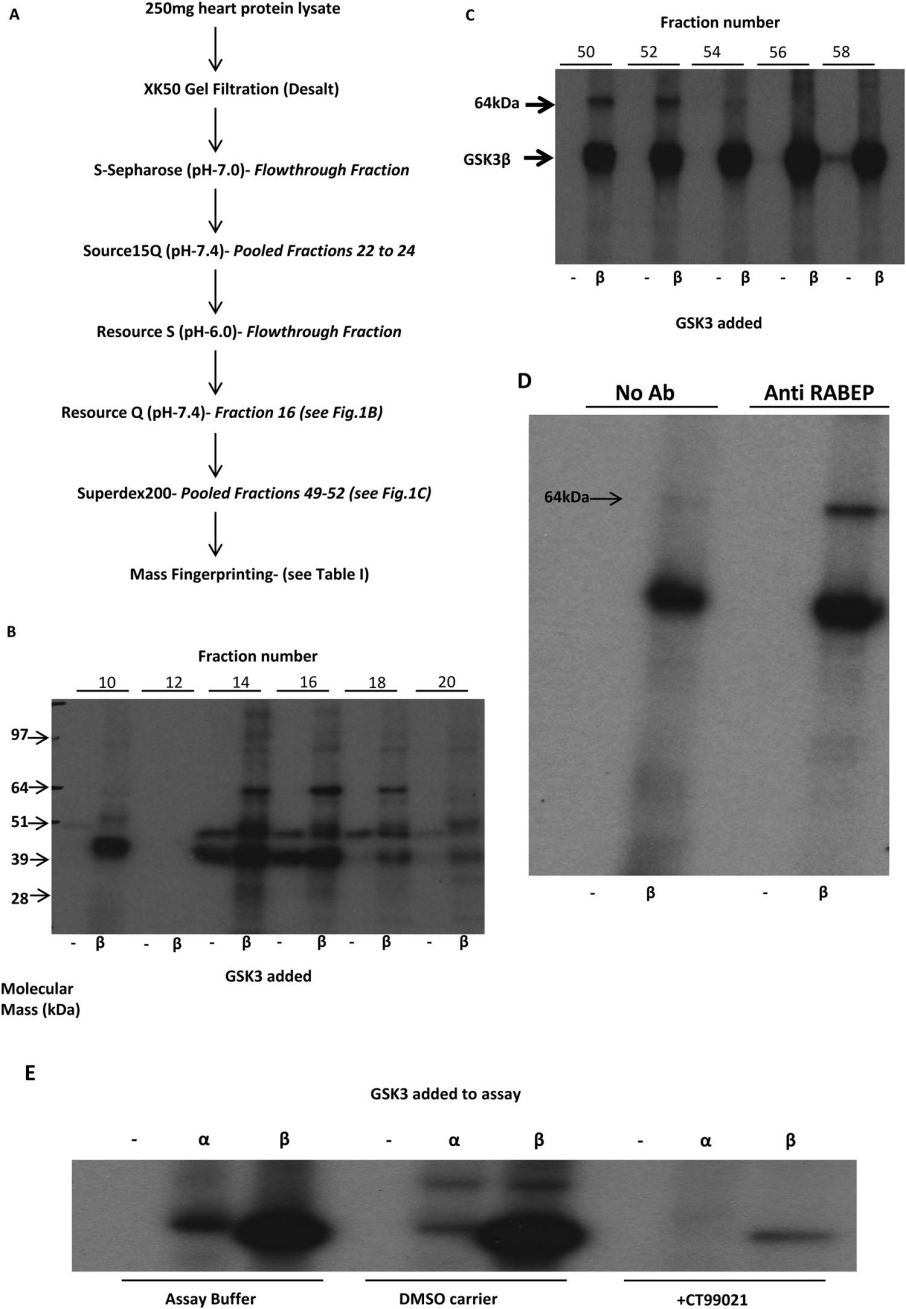
KESTREL screen identifies RABEP2 as a novel GSK3 substrate (**A**) Flow chart depicting purification steps of the KESTREL screen, with the fractions taken forward to the next step given in italics. (**B**) The flowthrough fraction from the Resource S (pH 6.0) column was loaded onto a Resource Q column and bound proteins eluted with a linear salt gradient to 1 M NaCl. Alternate fractions were incubated with (β) and without (−) 30 mU GSK3β and [γ-^32^P]-ATP for 5 min. Reaction proteins were separated by SDS-PAGE and radiolabelled proteins were visualised by autoradiography. (**C**) Fraction 16 from the Resource Q column was chromatographed on a Superdex 200 column and alternate fractions were incubated with (β) and without (−) 30 mU GSK3β and [γ-^32^P]-ATP for 5 min. The reaction proteins were separated by SDS-PAGE and visualised by autoradiography. (**D**) A pool of fractions 49–52 from the Superdex 200 column was incubated with (β) and without (-) 30mU GSK3β and [γ-^32^P]-ATP for 5 min before immunoprecipitation with an anti-RABEP2 antibody linked to agarose beads or beads alone (no Ab). Immunoprecipitated proteins were subjected to SDS-PAGE and visualised by autoradiography. (**E**) The pool of fractions 49–52 from the Superdex 200 column was incubated with (α or β) and without (-) 30mU GSK3α or GSK3β, and [γ-^32^P]-ATP for 5 min, then subjected to SDS-PAGE and visualised by autoradiography.

A KESTREL (Kinase substrate tracking and elucidation^16^) screen was performed using rat heart protein lysate to identify novel GSK3 substrates from this key metabolic tissue (where genetic reduction in GSK3 isoforms has pathophysiological outcomes^6,17^), as outlined in Fig. 1A. After each purification step an aliquot of each fractionated protein sample was incubated with and without active GSK3 and radiolabeled ATP before subjecting to SDS-PAGE and visualising phosphorylated proteins by autoradiography. A radiolabeled protein with an apparent molecular mass of 64 kDa was identified in fractions 14–18 eluting from the Resource Q anion exchange column (240–280 mM NaCl, Fig. 1A,B). This phosphoprotein only appeared when GSK3 was included in the incubation suggesting it was a target of GSK3 (Fig. 1B). The fraction containing the strongest signal (fraction 16) was further purified by size exclusion chromatography. Fractions 49–52 from the Superdex 200 column (representing molecular size >300 kDa) contained the 64 kDa GSK3 substrate (Fig. 1C), suggesting it is part of a large complex that dissociates upon SDS-PAGE. These fractions were pooled, subjected to SDS-PAGE and the 64 kDa protein was excised, digested with trypsin and released peptides identified by mass spectroscopy. Peptide sequences from eight proteins were identified but only one of these had a predicted molecular mass close to 64 kDa, namely RabGTPase Binding effector 2 (RABEP2, also termed rabaptin-5β, Uniprot Acc. No. Q9H5N1). Twelve separate peptides covering 22.38% of RABEP2 sequence were positively confirmed (Table 1). RABEP2 is a potential Rab5 regulatory protein first identified in a yeast 2-hybrid screen for Rab5 interacting proteins and is thus proposed to modulate vesicle trafficking^18^. RABEP2 has 42% sequence homology with rabaptin-5 (RABEP1) and is predicted to contain a C-terminal Rab5-binding domain plus an N-terminal Rabex-5-binding domain.

**Table 1:** Peptide sequences isolated from purified fraction containing a GSK3 substrate.

**PEPTIDES**	**Ion Score**	**Modifications**	**MH + (Da)**
LSQALQVR	63		914.54137
AEELIQEIQR	52		1228.65203
TLQGTVSQAQER	50		1317.67632
AQLTDLLSEQR	49		1273.67290
aAAPAALALDPQPQEEQKDASESSELSR	42	N-Term(Acetyl)	2951.41953
lSQALQVR	40	N-Term(Acetyl)	956.55083
qADTLEQVR	40	N-Term(Gln->pyro-Glu)	1042.51555
lQAELETSEQVQR	39	N-Term(Acetyl)	1572.78472
SILDEAPLR	37		1013.56102
qPASLHGSTELLPLSR	35	N-Term(Gln->pyro-Glu)	1688.89714
ASLEGQLR	33		873.47728
QADTLEQVR	32		1059.54192

Fractions 49–52 of the Superdex column were pooled, subjected to SDS-PAGE and visualised by colloidal coomassie blue staining. A gel piece representing 60–70 kDa Mass size was extracted from the gel, digested and analysed by HPLC linked to Peptide Mlass Fingerprinting. Twelve peptides were identified that confirmed the presence of RABEP2 in the fraction and their sequences are shown.

To confirm that the 64 kDa radiolabeled band was indeed RABEP2 we incubated an aliquot of the pool of fractions 49–52 (Fig. 1C) with GSK3 and radiolabeled ATP once more, then added either anti-RABEP2 antibody bound to protein A agarose beads, or beads alone (Fig. 1D). The radiolabeled 64 kDa protein was immunoprecipitated only when anti-RABEP2 was included (Fig. 1D). This strongly argues that the screen has identified RABEP2 as a substrate of GSK3. Finally, we found that the protein in the fraction was a substrate for both GSK3 isoforms (Fig. 1E).

### GSK3 phosphorylates RABEP2 at Ser200 and this is greatly enhanced by priming at Ser204

**Figure 2:**
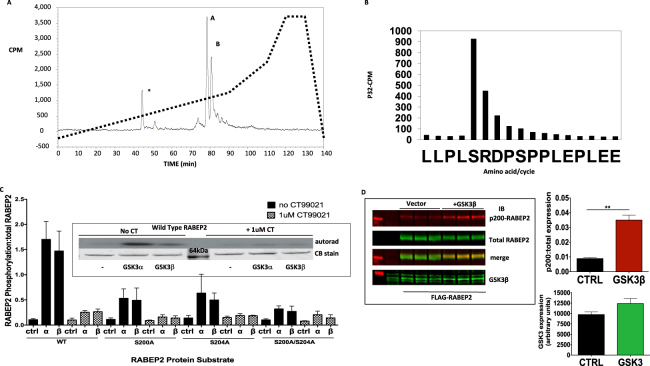
GSK3 phosphorylates RABEP2 at Ser200 following priming at Ser204. Immunoprecipitated FLAG-RABEP2 was incubated with 30mU GSK3β and [γ-^32^P]-ATP for 60 min prior to SDS-PAGE. (**A**) The gel piece containing the radiolabeled RABEP2 was digested with Glu-C and released peptides subjected to reverse phase HPLC. (**B**) Aliquots of the radiolabeled peptides (**A**) and (**B**) from Fig. 2A were analysed by Edman Degradation linked to gas chromatography and scintillation counting. The amino acid released at each cycle is given on the x-axis and the radioactivity released provided on the y-axis. (**C**) FLAG-RABEP2 WT, Ser200Ala, Ser204Ala or Ser200/204Ala mutants were expressed in HEK293 cells, immunoprecipitated and incubated *in vitro* with 30mU GSK3α or GSK3β or no GSK3 (Ctrl), and [γ-^32^P]-ATP ± 1μM CT99021(GSK3 inhibitor) for 60 min prior to SDS-PAGE and autoradiography. A representative autoradiograph (upper panel cropped at 64 kDa marker) with associated Coomassie Stain (lower panel) for the WT construct is provided as an insert while data from two separate experiments is provided in the graph (ratio of RABEP2 phosphorylation (CPM) normalized to densitometric quantification of the RABEP2 in every incubation, average ± SEM). (**D**) HEK293 cells were co-transfected with expression vectors for WT-RABEP2 plus either GSK3β or vector control. Cell lysates were prepared 24 h later, and these were subjected to Western blot analysis using the antibodies as indicated (upper panel). Representative images from one of two similar experiments are shown, while the graphs in the lower panel provide quantification of these experiments (**p < 0.001, t-test).

In order to identify the residues on RABEP2 phosphorylated by GSK3 we cloned and expressed the human FLAG-tagged RABEP2 in HEK293 cells. The cells were subjected to 3h serum starvation followed by 1h incubation with the GSK3 inhibitor CT99021 prior to cell lysis. As GSK3 substrates often require prior phosphorylation (priming) by a distinct kinase the aim was to block the phosphorylation of RABEP2 by GSK3 in the cells but maintain any priming of the ectopically expressed RABEP2. The FLAG-RABEP2 was immunoprecipitated and incubated with GSK3 and radiolabeled ATP. GSK3 introduced around 0.1 mole/mole phosphate into RABEP2 within 30 mins. The radiolabeled RABEP2 was subjected to SDS-PAGE, prior to in gel digestion of the RABEP2 band with Glu-C. The released peptides were separated by reverse phase HPLC and three radiolabeled peaks were observed using in-line scintillation detection (Fig. 2A). Two phosphopeptides (marked as peaks A and B in Fig. 2A) accounted for nearly 90% of the radioactivity detected and Mass Spectroscopy established that both peaks contained a peptide covering residues 196 to 212 of RABEP2 (Table 2, LLPLSRDPSPPLEPLEE). Peptide A was a diphosphopeptide (LLPLsRDPsPPLEPLEE, underlined residues phosphorylated), and peptide B was the monophosphopeptide version of the same sequence (LLPLsRDPSPPLEPLEE). Traces (around 6%) of the diphosphopeptide were seen in peptide B (Extracted Ion Chromatogram, data not shown). Aliquots of phosphopeptides A and B were subjected to solid phase Edman degradation and the radioactivity released at each cycle quantified (Fig. 2B). Radioactive phosphate was present only at the position equivalent to Ser200 (Edman cycle 5) in both the di- (Table 2) and mono- (data not shown) phosphopeptides, indicating this to be the site phosphorylated by GSK3 *in vitro* (labeled by ^32^P). This residue lies in a perfect consensus sequence for GSK3 where priming would occur at Ser204 (Table 2), the implication being that the other phosphate in the diphosphopeptide (Ser204) was thus phosphorylated prior to isolation from the HEK293 cells (and hence not radioactive). This is consistent with priming at Ser204 in the cells enhancing subsequent phosphorylation by GSK3 at Ser-200 *in vitro*. However Peak B was a singly phosphorylated peptide (at Ser200) suggesting that GSK3 can phosphorylate RABEP2 at Ser200 to some extent without priming, at least when incubated at high concentration *in vitro*. It is difficult to say from this data what the relative affinity of primed and unprimed phosphorylation may be as we do not know to what extent the ectopically expressed RABEP2 used in this experiment is primed in the HEK293 cells. The third phosphopeptide (* in Fig. 2A, accounting for 12% of the total peak radioactivity) was unidentifiable.

**Table 2:** Sequences of peptides radiolabeled by GSK3.

HPLC Peak	m/z	M (Expt)	M (Theor.)	Mascot Score	Sequence (aa 196–212)
**A**	1031.4820	2060.9494	2060.9472	38	E.LLPLsRDPsPPLEPLEE.L + 2Phospho
**B**	991.4981	1980.9816	1980.9809	61	E.LLPLsRDPSPPLEPLEE.L + Phospho
**B**	1031.4818	2060.9490	2060.9472	48	E.LLPLsRDPsPPLEPLEE.L + 2Phospho

The fractions from the HPLC column in Fig. 2A were analysed by in-line scintillation counting and radioactive fractions were subjected to Mass Fingerprinting for sequence detection. “s” in the Sequence indicates a Phosphorylated Serine residue. For the Mascot Score, an individual ion score of >21 indicates identity or extensive homology (p < 0.05). For the Mono-Phospho peptide; the Mascot delta score is >99.4% and the phosphoRS probability score is 100% respectively for S200 being PhosphoSerine. Peptide marked as * in Fig. 2A was unidentifiable.

In order to distinguish the role of priming at Ser204 more clearly we expressed Ser200Ala, Ser204Ala and Ser200/204Ala RABEP2 mutants in HEK293 cells, immunoprecipitated and incubated them with each GSK3 isoform as described for Fig. 2A above. Immunoprecipitated wild-type RABEP2 was phosphorylated by both GSK3 isoforms while inclusion of the small molecule GSK3 inhibitor CT99021 in the assay blocked phosphorylation (Fig. 2C insert). The phosphorylation of RABEP2 by GSK3 was equally blunted by mutation of Ser200, Ser204 or Ser200/204, while all mutations reduced phosphorylation to a similar extent to that seen by GSK3 inhibition using CT99021 (Fig. 2C). This confirms that Ser200 is the major GSK3 phosphorylation site and that priming at Ser204 greatly enhances phosphorylation of RABEP2 by GSK3. Finally, we found that co-expression of GSK3β with WT-RABEP2 increased the phosphorylation at Ser200 (Fig. 2D), indicating that enhancing GSK3 activity induces phosphorylation of this residue. This implies that enhanced priming at Ser204 may not be completely required to permit some enhanced phosphorylation of Ser200.

### RABEP2 is phosphorylated at Ser200 and Ser204 in cells

**Figure 3:**
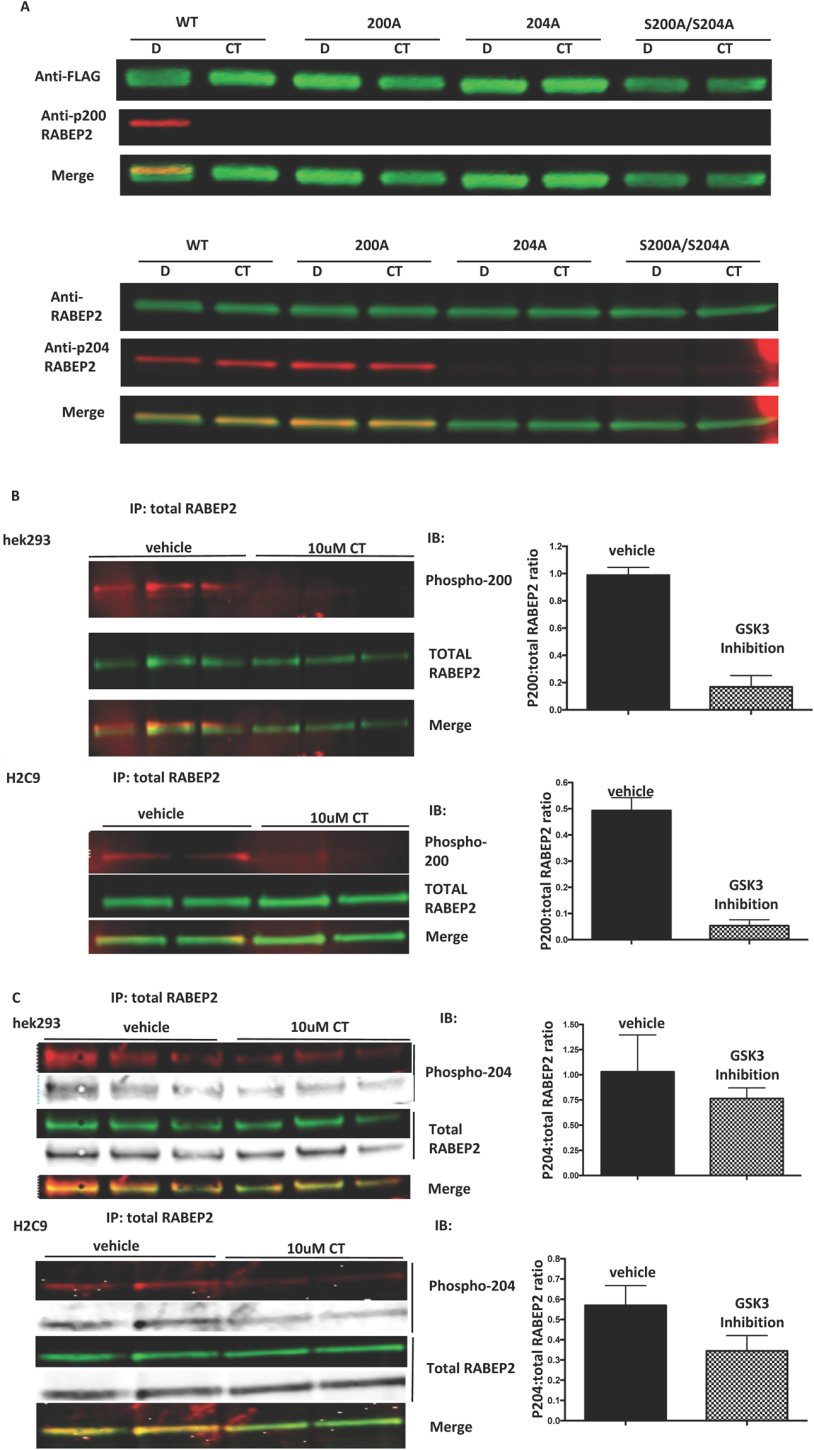
RABEP2 is phosphorylated in intact cells by GSK3. (**A**) FLAG-RABEP2 WT, Ser200Ala, Ser204Ala or Ser200/204Ala mutants were expressed in HEK293 cells which were then incubated ± 10 μM CT99021 (GSK3 inhibitor, CT) for 1 h prior to cell lysis. Protein lysates were immunoblotted with the antibodies as indicated. (**B**) and (**C**) HEK293 or H9C2 cells were incubated ± 10 μM CT99021 (GSK3 inhibitor, CT) for 1 h prior to cell lysis. Endogenous RABEP2 was immunoprecipitated from cell lysates and immunoblotted with the indicated antibodies. A representative image of two separate experiments performed in at least duplicate are provided. Quantification was performed using the Licor Odyssey software and the ratio of phosphor to total RABEP2 is provided in the graphs to the right of each figure. In all parts of the figure the fluorescent images were cropped between 60–70 kDa (original images of complete gels are provided in Supplementary Information), and the separate red 700 nm or green 800 nm Licor Odyssey scan (from same gel) plus the merged image are provided. In C the greyscale image of the same gel is also provided for clarity.

Human wild-type, Ser200Ala, Ser204Ala or the double Ser200/204Ala proteins were expressed in HEK293 cells, the cells incubated ± CT99021 to inhibit endogenous GSK3, and protein lysates isolated. Each lysate was probed with phosphospecific antibodies generated in-house to peptide sequences equivalent to those around Ser200 or Ser204 (see Methods). All RABEP2 constructs expressed to similar levels in the HEK293 cells (FLAG or total RABEP2 staining, Fig. 3A). However only wild-type RABEP2 was detected by the phospho-Ser200 antibody, while incubation of the cells with CT99021 (GSK3 inhibitor) prior to protein isolation abolished detection by this phosphospecific antibody (Fig. 3A). This suggested that GSK3 phosphorylated RABEP2 at Ser200 in human cells. In addition, the Ser204Ala mutant was not detected by the phospho-Ser200 antibody implying that the phosphorylation of Ser200 in cells required prior phosphorylation of Ser204. When we probed lysates with anti-phospho-Ser204 we could detect the wild-type and the Ser200Ala proteins but not the Ser204Ala mutant (Fig. 3A). This confirms that the antibody specifically detects phosphoSer204 and, consistent with our Mass Spectroscopy data (which identified non-radiolabeled phosphate in Ser204 (Fig. 2)), indicates that Ser204 is phosphorylated in cells. Importantly, incubation of cells with CT99021 did not reduce Ser204 phosphorylation confirming that GSK3 does not phosphorylate Ser204 in cells and that the anti-phosphoSer200 antibody does not detect phosphoSer204 (Fig. 3A).

Next, we immunoprecipitated endogenous RABEP2 from protein lysates generated from two different cell lines (HEK293 and the cardiomyocyte line H9C2) incubated ± CT99021 for 60 mins, and probed them with anti-RABEP2, anti-phospho-Ser200 (Fig. 3B) and anti-phospho-Ser204 (Fig. 3C). Endogenous RABEP2 was isolated from both cell lines and this was at least partially phosphorylated at Ser200 and Ser204. In line with the results with recombinant RABEP2, in both cases incubation of the cells with a selective GSK3 inhibitor reduced Ser200 but not Ser204 phosphorylation of endogenous RABEP2. This indicates that endogenous human RABEP2 is phosphorylated at Ser200 by GSK3 in cells.

### RABEP2 phosphorylation and function

**Figure 4:**
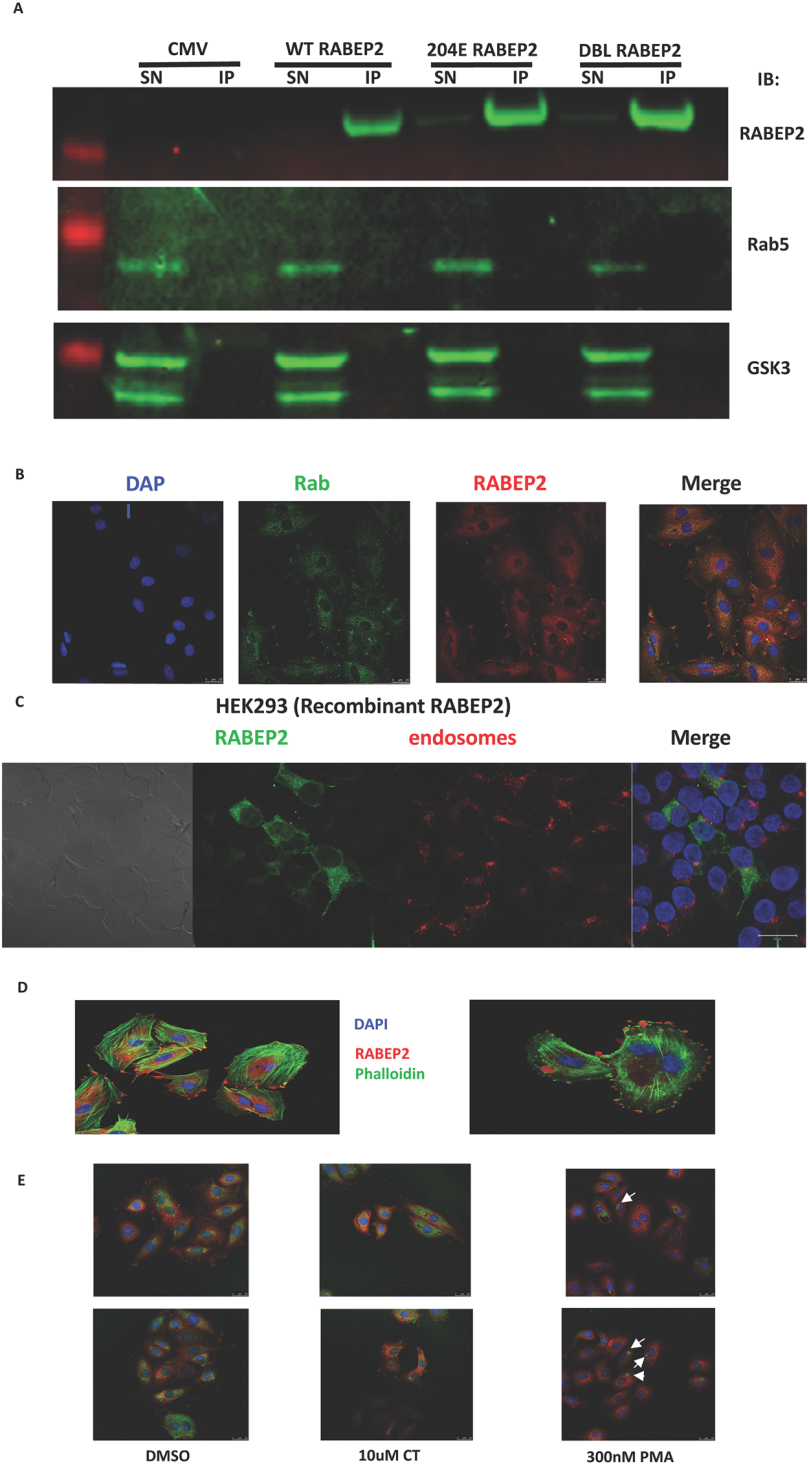
RABEP2 does not associate with Rab5, early endosomes or macropinosomes. (**A**) HEK293 cells were transfected with Flag-tagged-Wild-type (WT), Ser204Glu (204E) or Ser200Ala/Ser204Ala (DBL) RABEP2 expression vectors. Cell lysates were prepared 24 h later and 0.5 mg subjected to immunoprecipitation with anti-FLAG. The entire immunoprecipitate (IP), and 7.5% of the remaining supernatants (SN), underwent SDS-PAGE and transfer to nitrocellulose. The membrane was cut into three by molecular mass and the upper section probed with anti-total RABEP2, the middle section with anti-GSK3 and the lower with anti-rab5 antibodies (original images of membrane sections are provided in Supplementary Information). (**B**) H9C2 cells were co-stained for endogenous Rab5 (green) and endogenous RABEP2 (red) along with DAPI staining of nuclei. Channels are shown individually and after merging images. The average Pearson’s coefficient for 26 different images was 0.219 ± 0.143, suggesting little, if any, co-localisation. (**C**) GFP-tagged WT RABEP2 (green) was overexpressed in HEK293 cells and cells loaded with Transferrin-Alexa Fluor594 (red) to label early endosomes. Channels are shown individually and after merging images. (**D**) To image F-actin in the cytoskeleton H9C2 cells were incubated for 5 mins in PBS containing 0.33 uM (final) AlexaFluor488 Phalloidin (Green), and counterstained for RABEP2 (red). Coverslips were visualised using a Leica SP5 laser scanning confocal microscope. Two representative images are provided. (**E**) H9C2 cardiomyoblasts were serum starved for 3 hours before incubation with DMSO vehicle, PMA or CT99021 for 1 hr as indicated. FITC-dextran (green) was added to the cells to label macropinosomes, endogenous RABEP2 was visualised by immunofluorescence (red), and nuclei were stained using DAPI. Two representative images for each condition are shown. The data indicates that GSK3 inhibition (CT) does not cause internalisation of FITC-dextran into macropinosomes, while the PKC inhibitor (PMA) is a known inducer of macropinocytosis (arrowheads indicate macropinosomes). These very distinct intracellular structures do not co-stain with RABEP2 implying that RABEP2 is not localised onto macropinosomes.

RABEP2 was first isolated as a Rab5 interacting protein and the physiological function of RABEP2 is proposed to be the regulation of Rab5 mediated early endosomal recycling^18^. Unfortunately, immunoprecipitation experiments in the H9C2 cells did not identify a Rab5-RABEP2 interaction (Fig. 4A for example). It is noteworthy that there has been very little follow up work on the RABEP2 regulation of Rab5 since the original report. In addition, we could not identify RABEP2 associating with Rab5 in early endosomes of H9C2 cells (Fig. 4B) or HEK293 cells (data not shown) by immunofluorescence, while ectopically expressed RABEP2 did not co-localise with early endosomes in HEK293 (Fig. 4C) and had no effect on early endosomal recycling (data not shown). Therefore, establishing the role of phosphorylation on a proposed RABEP2 function has proven challenging. During immunolocalisation studies we did observe unusual staining of endogenous RABEP2 that had some resemblance to macropinosomes, lysosomes and membrane ruffles. However counterstaining with phalloidin (Fig. 4D), lysotracker (data not shown) or FITC dextran (Fig. 4E) indicated no co-localisation with ruffles, lysosomes or macropinosomes respectively. In addition, the GSK3 inhibitor CT99021 had no effect on macropinocytosis, compared with the known regulation by PKC activation (PMA) (Fig. 4E). The investigation of the impact of phosphorylation of RABEP2 will require more information on the physiological (and pathophysiological) role of this poorly studied protein.

## Discussion

Preclinical work suggests that reducing GSK3 activity in diseases such as diabetes, cancer and Alzheimer’s Disease could have major health benefits^19–23^, with the most compelling evidence from animal models where partial deletion can abrogate disease progression and/or initiation^3,10–12^. That said, isoform specific reduction may have both positive and negative effects on different aspects of health^6,17^, while complete deletion of either of the two GSK3 genes in rodents has serious consequences to the health of the animals. Therefore, we believe that improving our understanding of the upstream regulatory pathways and downstream targets that mediate the control and function of GSK3 isoforms in health, and importantly in disease states, may provide more acceptable ways to manipulate GSK3 for clinical benefit, as opposed to simply contemplating complete ablation of GSK3 activity. Here we describe identification of a novel GSK3 substrate, RABEP2, and show that GSK3 phosphorylates Ser200 of RABEP2 following priming at Ser204 (numbering relates to human sequence). We provide evidence using site-specific antibodies and Mass Spectrometry that these residues are phosphorylated in cells (both for endogenous or ectopically expressed protein), while previous global phosphoproteomic studies have also found that these residues on endogenous RABEP2 are phosphorylated in various cell systems (www.phosphosite.com). The residues are conserved throughout mammalian species, however they are not present in the related protein RABEP1. Importantly incubation of cells with a selective GSK3 inhibitor reduces Ser200 phosphorylation of ectopically produced and endogenous RABEP2. Clearly it is important to know whether this phosphorylation regulates RABEP2 function, but there is also a lack of good clinical biomarkers of GSK3 activity and thus it will be useful to establish if this phosphorylation event could be used as part of a biomarker panel for dysregulation of GSK3 action in different human diseases (or as a marker for quantifying GSK3 manipulation in clinical trials of drugs aimed at the PI3K-Akt-GSK3 pathway).

**Figure 5:**
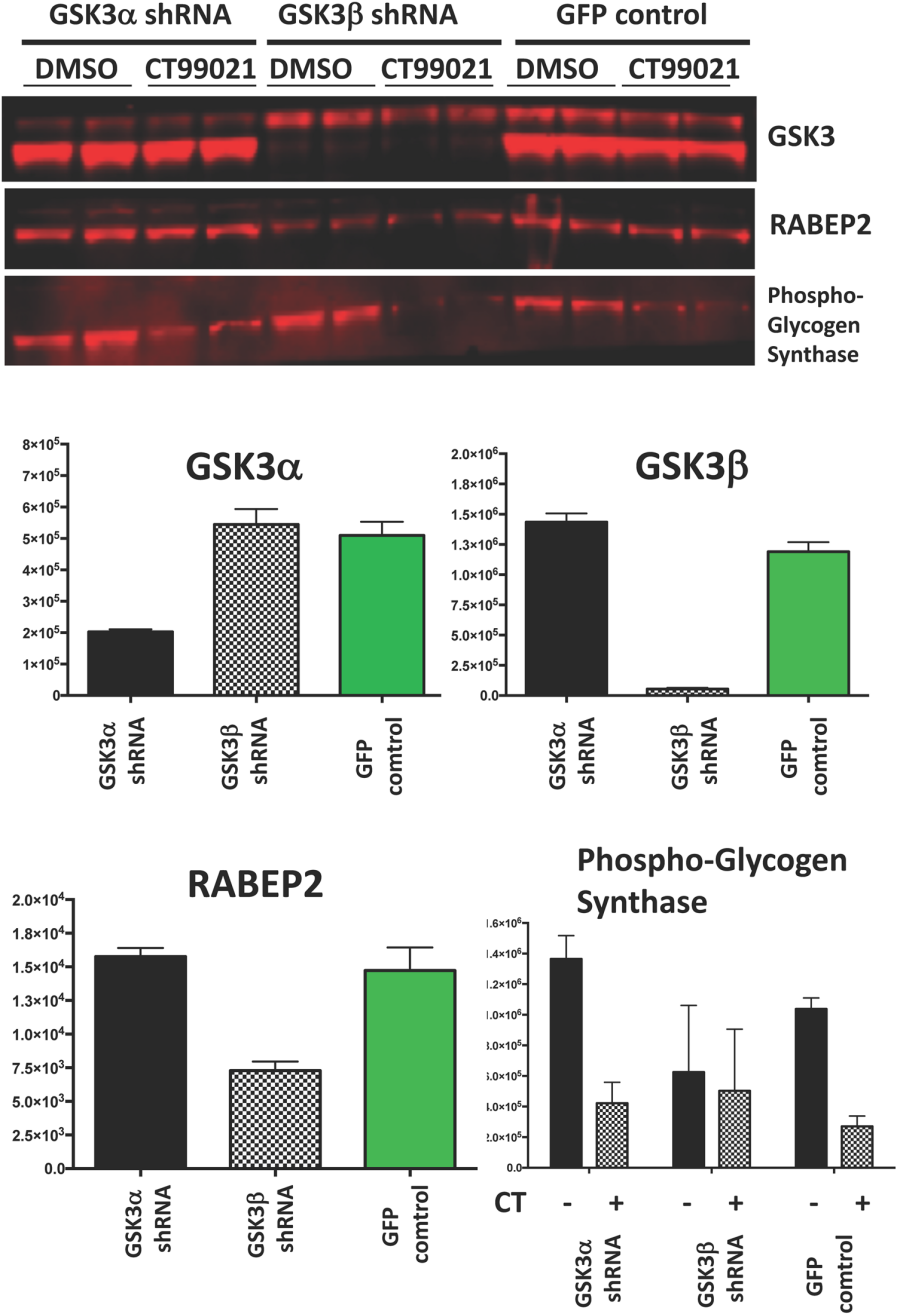
Knockdown of GSK3β reduces endogenous RABEP2 production in H9C2 cells. H9C2 cells had shRNA constructs for each GSK3 isoform inserted using a lentiviral stable expression system (Sigma Mission particles). Clones with robust reduction in GSK3 isoform protein production were isolated and representative lines, along with a GFP expressing line generated at the same time, were treated with or without 10 μM CT99021 or DMSO carrier for 1 h. Cell lysates were probed with the antibodies as indicated.

RABEP2 was first isolated in a yeast 2-hybrid screen for Rab5 binding proteins^18^. Rab5 is a well studied signaling protein, key for metabolic processes and is best known as a regulator of early endosome biology^24^. Early endosomes of the endocytic pathway are intracellular, protease-rich environments vital for the proper internalization, recycling, and catabolic modulation of various macromolecules. Rab5 depletion reduces the number of early endosomes, late endosomes and lysosomes, is associated with a block of low-density lipoprotein endocytosis, and produces a defect in polarity of the liver which severely abrogates the delivery of apical proteins to the bile canaliculi^24^. RABEP2 is reportedly recruited to the endosomal membrane by Rab5 in a GTP-dependent manner where it forms a complex with Rabex-5, the GDP/GTP exchange factor for Rab5. Complete early endosome fusion is proposed to require the presence of both Rabaptin-5 (RABEP1) and RABEP2 complexes, suggesting Rab5 signaling encompasses at least two distinct but cooperative complexes for optimal endocytotic membrane docking and fusion^18^. Importantly depletion of RABEP2 only partially modulated endosomal function, and to a much lower extent than that achieved by reduction of RABEP1^18^. Interestingly GSK3 can also be found in multivesicular endosomes^25^, and thus the literature supported a model where GSK3 could phosphorylate RABEP2 within endosomes thereby modulating the functional interaction between RABEP2 and Rab5. However, we were unable to generate any evidence from immunofluorescence, immunoprecipitation or endosomal function assays to support the co-localisation of Rab5, RABEP2 and endosomes. In addition, we did not find any modulation of endosomal recycling following ectopic expression of RABEP2. Our immunfluorescence experiments indicated some unusual staining patterns for RABEP2 (endogenous and ectopic), quite distinct from endosomes, and these did vary between cell types and with cell treatments. For example, in Fig. 4 the staining of RABEP2 in H9C2 cells in particular consistently highlighted membrane associated structures, as well as punctate cytoplasmic staining. In contrast, when the cells were serum starved, the cytoplasmic staining became more apparent (Fig. 4E). Our pilot counter staining experiments suggest the structures do not appear to be macropinosomes, lysosomes or membrane ruffles (Fig. 4D,E). The work could suggest that the regulation of Rab5 may not be the major, or generic, function of RABEP2. Even in the original work demonstrating Rab5 interaction the regulation of endosomal function by RABEP2 was relatively weak, and supports the likelihood of Rab5-independent cellular functions of RABEP2. It is noteworthy that other reported Rab5 regulatory proteins have subsequently been found to target distinct Rab proteins^26^. In short the lack of a robust RABEP2 functional assay makes it very challenging to establish the role of the phosphorylation by GSK3. Ultimately it will require more generic approaches to establish the key physiological functions of RABEP2, and as we learn more about this protein it will be possible to establish the role of phosphorylation by GSK3 on these potential functions. Indeed more recent work has identified RABEP2 in a screen for proteins that modulate the function of cilia^27^, while others have reported RABEP2 as a contributor to collateral blood vessel development (implicating RABEP2 as a potential modulator of severity of stroke and cardiac ischemia)^28^. Further recent work, assessing the GSK3 regulated proteome, reliably detected RABEP2 in WT ES cells but could not detect this protein in GSK3 double knockout (DKO) ES cells^29^. Importantly, there was no difference in the RABEP2 mRNA abundance between the WT an DKO cell lines. This implies that GSK3 activity stabilizes the RABEP2 protein, at least in ES cells. Of course, this does not prove that stabilisation of RABEP2 requires a direct phosphorylation by GSK3, but it represents clear evidence linking GSK3 activity and RABEP2 function. We have observed a similar reduced expression of endogenous RABEP2 in H9C2 cells stably expressing GSK3β (but interestingly not GSK3α) shRNA (Fig. 5). In contrast, WT RABEP2 and Ser200Ala/Ser204Ala double mutants were expressed to similar levels in HEK293 cells, while endogenous RABEP2 levels were not significantly altered in cells treated with a GSK3 inhibitor for up to 24h. This suggests that any regulation of RABEP2 protein abundance by GSK3 may not be easily detected in gross overexpression studies (it is possible that the degradation rate is limiting). Most likely the generation of Ser204Ala and Ser200Ala RABEP2 knockin animal models is required to conclusively demonstrate the role of RABEP2 phosphorylation on its function, although this may result in a RABEP2 null, if the phosphorylation is required for long term stabilization of endogenous RABEP2.

Finally, we propose that RABEP2 phosphorylation assessment could be a useful biomarker for GSK3 activity in disease, and after interventions to correct PI3K-Akt-GSK3 pathway dysfunction. Our data clearly shows that the Ser-200 phosphorylation of RABEP2 occurs in intact cells and is lost when cells are treated with a GSK3 inhibitor. Very few proposed GSK3 substrates have been validated in this way and thus the phospho-specific antibodies that we have generated may prove a useful tool for monitoring GSK3 status in disease models and in clinical trials.

In summary, we have identified a novel substrate for GSK3, elucidated a key role for priming in its regulation, established the site phosphorylated by GSK3, and confirmed that this is a physiological GSK3 target in cells. Establishing the effect of phosphorylation on RABEP2 function will require more detail on the physiological function of this poorly studied protein. However, this work provides the platform to investigate the role of RABEP2 phosphorylation on such functions as they are identified, and may provide a rationale for the recent report that RABEP2 protein (and not mRNA) is absent from ES cells lacking GSK3 activity. We would like to add RABEP2 to the list of potential GSK3 substrates linking GSK3 dysfunction to human disease.

## Experimental Procedures

### Materials

CT99021 (CAS number **252917-06-9**) is a highly selective, ATP competitive inhibitor, of GSK3 and was synthesised in-house as described previously^30,31^. [γ^32^P]-ATP was obtained from Perkin Elmer (Waltham, MA), Whatman GD/X filters and all FPLC columns were from GE Healthcare (Chalfont, Bucks, UK). All cell culture media was from Lonza (Basel, Switzerland). General chemicals were purchased from Sigma or Fisher Scientific and were of the highest grade available. Active GSK3α was purchased from Millipore (Billerica, MA, USA). GSK3β was produced in baculovirus by the Division of Signal Tranduction and Therapy, University of Dundee. Wheat Germ expressed GST-tagged RABEP2 was purchased from Abnova (Taipei, Taiwan).

### Commercial Antibodies

Rabbit anti-RABEP2 (Cat. No. 14625-1-AP) was purchased from Proteintech (Chicago, USA), Rabbit Anti-Rab5 was from AbCam (Cat. No. Ab18211), mouse anti-M2-FLAG (Cat. No F3165) was from Sigma Aldrich (Poole, UK), Rabbit anti-pGSK3 (S9/21) (Cat. No. 8566), and Rabbit anti-GSK3 α/β(Cat. No. 9315), Rabbit anti-phospho-glycogen synthase (Ser641) (Cat. No. 3891), were all from Cell Signaling Technologies. Anti-mouse Alexa Fluor 594 (Cat. No. A11032) and Alexa Fluor 680 (Cat. No – A21102) were from Thermo Fisher (Paisley, UK), and IR800CW (Cat. No – 611-131-002) was from Rockland Immunochemicals (Gilbertsville, PA).

## Methods

### Cell Culture

HEK293 cells were cultured in DMEM containing 4.5 g/l glucose and no sodium pyruvate and supplemented with 10% FCS and 1% penicillin/streptomycin. H9C2 rat myoblast cells were cultured in DMEM containing 4.5 g/l glucose and sodium pyruvate and supplemented with 10% FCS and 1% penicillin/streptomycin. All cells were subcultured to maintain confluency between 50 and 70%.

### Immunoprecipitation

Protein A agarose beads (Fisher Scientific, Waltham, MA) were conjugated to either rabbit Anti-RABEP2 (4 μg per mg pre-cleared cell lysate), or mouse Anti-FLAG (1 μg per mg pre-cleared cell lysate), for 1 hour at room temperature with shaking. Excess antibody was removed by washing pelleted beads in lysis buffer, and then beads added to cell lysates before incubation overnight at 4 ºC with shaking. Immunocomplexes were pelleted and washed twice using either lysis buffer containing 0.5 M NaCl (for subsequent WB), or assay buffer containing 0.5 M NaCl (for subsequent kinase assay), followed by once in buffer without salt. Experiments to detect binding proteins (eg Rab5) were washed in lysis buffer (with no additional NaCl).

### Preparation of rat heart protein lysates

22 Sprague Dawley male rats (2–3 month old, Harlan UK) were euthanized by cervical dislocation and the hearts excised whilst still contracting. All experimental procedures were performed in accordance with UK Home Office regulations under the auspices of Project Licence PIL60/3766. No procedures in this work required ethical approval. Hearts were immediately immersed and flushed with ice cold buffer (130 mM NaCl, 5.4 mM KCl, 1.4 mM MgCl2, 0.4 mM NaH2PO4, 4.2 mM HEPES) before snap freezing in liquid nitrogen. Hearts were thawed at 4 °C and homogenized to a fine paste using a Moulinette yoghurt maker. Homogenate was transferred to a blender and homogenized in 2.5 w/v of 4 mM EDTA, 50 mM NaF, 5% (vol/vol) glycerol, 0.1% (vol/vol) Pefabloc, 0.1% (vol/vol) β-mercaptoethanol before centrifugation at 4000 rpm for 5 mins at 4 °C and the supernatant collected. Lysate was filtered through gauze, followed by a Whatman GD/X filter (Sigma, UK) before being snap frozen. 250 mg (50 ml of 5 mg/ml) of lysate was desalted into 30 mM MOPS pH7, 0.1 mM EGTA, 5% (vol/vol) Glycerol, 0.03% (vol/vol) Brij 35 on a XK50 gel filtration column.

### KESTREL (Kinase substrate tracking and elucidation)

The desalted heart lysate was fractionated by several rounds of protein purification^16^ (Fig. 1a). At each stage an aliquot of each fraction was incubated with or without GSK3β (30 mU), in Assay buffer (30 mM TRIS-HCl pH7.5, 0.1 mM EGTA, 0.1% (vol/vol) β-mercaptoethanol), along with 5 mM MnCl_2_ and 20 nM γ-^32^P-ATP (10^6^CPM/nmole 6000 mCi/ml) in a final volume of 31ul for 5 mins at 30 °C. Reactions were stopped by the addition of 10ul pre-warmed 4xLDS buffer (95 °C) containing 10 mM DTT and then left at 95 °C for 5 min before separation of constituents by 12% TRIS-Glycine SDS-PAGE on 16 cm × 16 cm ATTO AE-6220 gel system (Tokyo, Japan). Proteins were transferred onto PVDF membrane overnight using Bio-Rad Trans-Blot Plus system (Bio-Rad, Herts, UK) and radiolabeled proteins visualized by autoradiography. Fractions found to contain proteins with significant phosphorylation after incubation with GSK3 were further purified and re-assayed until judged sufficiently pure to analyse by Mass Fingerprinting. Protein bands were then excised, digested with trypsin and the released peptides dissolved in 1% formic acid prior to reverse phase HPLC (C18 column, 7.5 um, 50 cm) at a flow rate of 0.3 ul/min. The peptides were eluted from the column with a gradient from 2%B to 40%B in 65 min (Buffer A: 0.1% formic acid in water, Buffer B: 0.08% formic acid in 80% CH3CN) and then analysed on a LTQ-Orbittrap Velos Mass Spectrometer equipped with an easy spray source. First, a full MS survey scan at a resolution of 60 K was acquired in Oribttrap, then the fifteen most intense PRECURSOR ions were sequentially selected to be fragmented by LTQ, the multistage activation scan was trigged if a neutral loss of 97.9, 49.8, 32.7 and 24.5 were detected. The MS data were searched against IPIrat database through the proteome discovery software (1.4) using mascot search engine (Matrix Science, Version 2.2)). The search results were filtered using the following criteria: 1%FDR and a minimum of two peptides.

### Cloning, mutagenesis and protein expression

cDNA of full length human RABEP2 (accession number – NM_024816) was amplified by PCR using the primers detailed in Table 3A and cDNA isolated from SH-SY5Y neuroblastoma cells as template. PCR primers incorporated an N-terminal FLAG tag and EcoR1 restriction sites for subcloning. Human RABEP2 PCR product was first placed into pGEM T-easy vector system (Promega, Madison, WI), and sequenced before sub-cloning into either pGEX 6p-1 for bacterial expression or pCMV5 for mammalian expression. Mutagenesis of RABEP2 was performed by site directed mutagenesis using the primers detailed in Table 3B following the manufacturer’s protocol (Agilent, Santa Clara, CA). GST-RABEP2 fusion protein was produced in *E. Coli* by standard protocols, followed by purification on glutathione Sepharose. The GST-tag was removed by incubation of the fusion protein with GST-Precission Protease, and repurification on glutathione Sepharose.

**Table 3:** DNA Oligomers used in the molecular cloning of RABEP2.

**A) PCR primers**	**Sequence**
pGEX fwd	GAATTC **GACTACAAGGACGACGATGAC**GCGGCAGCTGCGCCGGTGG
pCMV fwd	GAATTC *GCCACC*ATG**GACTACAAGGACGACGATGAC**GCGGCAGCTGCGCCGGTGG
rev	GAATTCTCAGGTGTCCTTGATGTCCCTGACG
**B) Mutagenesis**	**Sequence**
S200A fwd	GTTGCTGCCCCTG**G**CCCGGGATCCATC
S200A rev	GATGGATCCCGGG**C**CAGGGGCAGCAAC
S204A fwd	CTGTCCCGGGATCCA**G**CGCCCCCG
S204A rev	CGGGGGCG**C**TGGATCCCGGGACAG
S204E fwd	CCCTGTCCCGGGATCCA**GA**GCCCCCGCT
S204E rev	AGCGGGGGC**TC**TGGATCCCGGGACAGGG

(A) Primer sequences used to generate hRABEP2 cDNA for subcloning into pGEX and pCMV expression vectors. EcoR1 restriction sites are underlined, a Kozac sequence is in italics, while the FLAG tag sequence is in bold. (B) Site directed mutagenesis primer sequences with the base changes introduced to produce amino acid modifications shown as underlined and in bold.

### Mammalian Cell Transfection

HEK293 cells were plated onto 10 cm^2^ dishes at a density of 5 × 10^5^ cells/ml and incubated at 37 °C, 5% CO2 overnight. 4 ug of DNA was transfected per dish using 30 ul Lipofectamine 2000 (Invitrogen, Paisley, UK) according to the manufacturer’s protocol. After 4 h, media was replaced with normal growth media, then incubated for a further 18 h prior to treatment and/or protein isolation.

### Phosphorylation site mapping

HEK293 cells were transfected with pCMV-FLAG-RABEP2 and 24 h later cells were serum starved for 3 h before incubation with 10 μM CT99021 for 1 h (to inhibit endogenous GSK3 and dephosphorylate GSK3 target sites). Cells were washed once with PBS before lysis in 300 ul lysis buffer (50 mM Tris-HCl pH7.4, 50 mM NaF, 1 mM Na-pyrophosphate, 1 mM EDTA, 1 mM EGTA, 50 mM NaCl, 1% triton X-100, 0.27 M sucrose, protease inhibitors, 0.1% β-mercaptoethanol, 1 mM Na_3_VO_4_). Protein lysates were spun at 13 K rpm, 4 °C for 10 min to remove insoluble material and protein concentration measured by Bradford Assay^32^. 1 mg of cell lysate was incubated overnight at 4 °C with 50 μg anti-FLAG conjugated to Protein G agarose beads (Sigma, Poole, UK). The FLAG-RABEP2 was precipitated by centrifugation washed twice in assay buffer (30 mM TRIS-HCl pH7.5, 0.1 mM EGTA, 0.1% v/v β-mercaptoethanol) containing 0.5 M NaCl followed by one wash in assay buffer. Beads were resuspended in 24 μl assay buffer and incubated for 1 h with 30 mU GSK3α or GSK3β plus 10 mM MgCl_2_ and 0.1 mM [γ-^32^P] ATP in a final volume of 30 μl. The assay was stopped and the FLAG-RABEP2 released from the beads by the addition of 4xLDS (with 10 mM DTT) and heating at 60 °C for 5 min. Supernatants were boiled to 95 °C for 5 min and allowed to cool on ice before alkylation of the protein by addition of iodoacetamide (to 50 mM) for 30 mins at RT in the dark. Proteins were separated by SDS-PAGE, stained using SimplyBlue SafeStain before visualization of radiolabeled protein by autoradiography. Labelled proteins were excised and the amount of ^32^P incorporated was estimated by Cerenkov counting. Gel pieces were completely destained by successive washes in water, 50% acetonitrile/water, 0.1 M NH_4_HCO_3_ 50% acetonitrile/50 mM NH_4_HCO_3_ (0.5 ml for 10 min at RT), and finally 50% acetonitrile/50 mM NH_4_HCO_3_ (overnight at 4 °C). The gel pieces were then dehydrated in 0.3 ml acetonitrile for 15 min at RT, dried, and incubated with 25 mM Triethylammonium bicarbonate (TEB) containing 5 ug/ml Glu-C protease with shaking at 30 °C overnight. An equivalent volume of acetonitrile was added and the digest continued for 15 min before removing the supernatant for freeze-drying. Gel pieces were resuspended in 100ul 50% acetonitrile/2.5% Formic acid with shaking for 10 min and the resultant supernatant, combined with the dried first extract, was freeze-dried prior to further purification. ^32^P radioactivity incorporated was recovered and these peptides were chromatographed on an Ultimate 3000 LC system (Thermo), equipped with a FlowStar LB 315 on-line radioactivity monitor (Berthold), utilising a Vydac 218TP5215 C_18_ column (Separations Group, Hesperia, CA, U.S.A.) equilibrated in 0.1% trifluoroacetic acid in water. The column was developed with a linear acetonitrile gradient (diagonal dashed line in Fig. 2a) at a flow rate of 0.2 ml/min and fractions of 0.1 ml were collected. Recovery of ^32^P from the column was around 85%.

### Mass Spectrometric Analysis

Mass spectrometric analysis of the Glu-C derived peptides was performed by LC-MS-MS on a linear ion trap-orbitrap hybrid mass spectrometer (Orbitrap-Classic, Thermo) equipped with a nanoelectrospray ion source (Thermo) and coupled to a Proxeon EASY-nLC system. Peptides were injected onto a Thermo (Part No. 160321) Acclaim PepMap100 reverse phase C_18_ 3μm column, 75μm × 15 cm, with a flow of 300 nl/min and eluted with a 45 min linear gradient of 95% solvent A (2% Acetonitrile, 0.1% formic acid in H_2_O) to 35% solvent B (90% acetonitrile, 0.08% formic acid in H_2_O) at 20 min followed by a rise to 80%B at 23 min, maintained at 80% B for 5 min, followed by re-equilibration. The instrument was operated with the “lock mass” option to improve the mass accuracy of precursor ions and data were acquired in the data-dependent mode, automatically switching between MS and MS-MS acquisition. Full scan spectra (m/z 340–1800) were acquired in the orbitrap with resolution R = 60,000 at m/z 400 (after accumulation to an FTMS Full AGC Target; 1,000,000; MSn AGC Target; 100,000). The 5 most intense ions, above a specified minimum signal threshold (5,000), based upon a low resolution (R = 15,000) preview of the survey scan, were fragmented by collision induced dissociation and recorded in the linear ion trap, (Full AGC Target; 30,000. MSn AGC Target; 5,000). Multi-Stage-Activation was used to provide a pseudo MS3 scan of any parent ions showing a neutral loss of 48.9885, 32.6570, 24.4942, allowing for 2+, 3+ and 4+ ions respectively. The resulting pseudo MS3 scan was automatically combined with the relevant MS2 scan prior to data analysis. RAW files containing data from the Orbitrap-Classic were analysed directly by using Proteome Discoverer 1.4 and phosphoRS 3.1 (Thermo), utilising Mascot (www.matrixscience.com), and searching against an in house database containing the relevant sequences.

### Edman Sequencing of ^32^P labelled Phosphopeptides

Performed as in Campbell & Morrice^33^ except that the Edman sequencing was performed on a Shimadzu PPSQ-33A system.

### Generation of phospho-specific antibodies to p-Ser200 and p-Ser204 of RABEP2

Peptides were synthesized based on the sequence surrounding Ser200 of RABEP2 [TELLPLS(Ph)RDPSPPL] (with residue equivalent to Ser200 chemically phosphorylated (Ph)), conjugated to Keyhole limpet hemocyanin and injected into a sheep. Antibodies were affinity purified from serum using the phospho-peptide antigen, and antibody released from the matrix by low pH. A similar protocol was used for the generation of p-Ser204 specific antibodies using the peptide [LSRDPS(Ph)PPLEPL]. The equivalent dephospho-peptides were synthesized and incubated with the phosphospecific antibodies prior to use in immunoblot to minimize background.

### Western Blotting

Protein extracts were separated on Novex SDS 4–12% polyacrylamide gels. Following transfer to PVDF, blots were blocked with 1% BSA (w/v) in TBST [Tris-buffered saline (20 mM Tris/HCl and 150 mM NaCl) containing 0.1% Tween 20] at RT for 30 mins, and then incubated with primary antibodies at 4 °C overnight, prior to incubation for 1 h at RT with appropriate secondary antibody and development using a Licor Odyssey two dye nearIR (green-800 nm/red-700 nm) Imaging System, which allowed phosphorylation (anti-sheep 2ry) and total protein (rabbit or mouse 2ry) images to be developed from the same blot and images to be merged (Fig. 3). All primary antibodies were diluted in 1% BSA/TBS-T; anti-total RABEP 2 diluted 1:2000, anti-FLAG diluted 1:5000, anti-phospho 200 RABEP2 diluted 1:250 and included 1 μM non-phosphopeptide, anti-phospho204 RABEP2 diluted 1:250 and included 1 μM non-phosphopeptide. All secondary antibodies were diluted in TBS-T at 1:1000. The phosphor-GSK3(S9/21) Western blots were carried out as above except primary antibodies were diluted 1:1000 and bands visualized by chemiluminescence using HRP-conjugated Anti-Rabbit secondary antibody.

### Generation of GSK3 knockdown H9C2 cells

H9C2 cells were infected with lentiviruses with expression cassettes to produce shRNA targeting either GSK3α or GSK3β (Sigma Aldrich Mission particles). Cells were infected in 6 well plates with one of 4 different shRNA viruses for each GSK3 isoform, or with GFP virus. Cells with stable insertion of virus were selected by survival in puromycin. Clone 10551 had almost complete ablation of GSK3β production, while clone 195223 had >60% reduction in GSK3α. The GFP control cells were used as the ‘normal expression’ for each GSK3 isoform. These three lines were serum starved for 3h, then incubated with either DMSO or CT99021 for 1h prior to cell lysis. The lysates (11μg) were probed by Western blot to examine effects on RABEP2, and the validated GSK3 substrate, glycogen synthase (phosphor GS-Ser641).

### Microscopy

#### Immunofluorescence

H9C2 cardiomyoblasts were plated onto coverslips and left overnight to attach. Cells were serum starved for 1 hour then 10μM CT99021 (GSK3 inhibitor) added and cells incubated at 37 °C for 1 hour prior to a wash with PBS and fixation in 4% PFA for 30 mins at RT. Cells were permeabilised with PBS containing 0.1% (vol/vol) Triton × 100. Non-specific binding was blocked by incubation with PBS-Tween containing 1% BSA before exposure to anti-RABEP2 (1:500) or anti-Rab5 (1:250) antibodies for 45 mins at RT. Coverslips were washed 3x in PBS containing 0.1% Triton X100 before addition of AlexaFluor594 (rabbit) or AlexaFluor488 (mouse) labelled secondary antibody (1:500) for 45 mins at RT in the dark. Excess antibody was removed by washing 3x PBS/0.1% Triton X100 before a final wash with PBS containing DAPI. To image F-actin in the cytoskeleton cells were incubated for 5 mins in PBS containing 0.33uM (final) AlexaFluor488 Phalloidin. Coverslips were mounted using Hydromount (National Diagnostics) and visualised using a Leica SP5 laser scanning confocal microscope at ×63 magnification. Pearson’s correlation coefficient was calculated using Volocity software (Perkin-Elmer). Thresholds were calculated automatically from the images.

#### Endosome staining

Briefly, HEK293 cells were transfected with GFP-tagged WT-RABEP2. After 24 hours, cells were trypsinised and plated onto coverslips in 6 well plates at a density of 5 × 10^5^ cells/well and allowed to attach to the coverslips overnight. Before assay, cells were incubated for 30 mins at 37 °C in serum free alpha minimal essential media (MEM) containing 0.5% BSA to remove any residual transferrin before incubation with 15ug/ml AlexaFluor 594 transferrin for 30 mins at RT in the dark. Media was removed and cells washed once with PBS before fixation in 4% PFA for 30 mins at RT. Coverslips were washed 3x PBS/0.1% Triton X100 before a final wash with PBS containing DAPI. Images were obtained on a Zeiss LSM700 confocal microscope (Carl Zeiss), with a ×100 alpha Plan-Apochromat objective (NA 1.43), using the 488-nm excitation laser, and the emissions were collected at 510–560 nm.

#### Macropinocytosis

H9C2 cardiomyoblasts were plated onto coverslips and left overnight to attach. Cells were serum starved for 3 hours before incubation with vehicle, 0.5ng/ml (final) PMA or 10uM CT99021 for 1 hr as indicated in the figure legends. FITC-dextran (100ug/ml final) was added to cells for 15 mins at 37°C to label macropinosomes. Cells were washed 3x PBS before fixation in 4% PFA for 30 mins at RT and permeabilisation with 0.1% Saponin. Non-specific binding was blocked using PBS-Tween/1% BSA. The RABEP2 antibody and the AlexaFluor594 anti-rabbit 2ry antibody were used at 1:500 dilution for 45 mins at RT in the dark. Nuclei were counterstained using DAPI (1:5000). Images were obtained using a Leica SP5 laser scanning confocal microscope at ×63 magnification.

## Electronic supplementary material

Supplementary Information
