# Supplementary material for: Rab-GTPase binding effector protein 2 (RABEP2) is a primed substrate for Glycogen Synthase kinase-3 (GSK3)
Source: Sci Rep. 2017 Dec 15;7:17682. doi: 10.1038/s41598-017-17087-6 (PMC5732219; doi:10.1038/s41598-017-17087-6)
Supplement: Supplementary file 1 — Supplementary Information [file 41598_2017_17087_MOESM1_ESM.pdf]

Rab-GTPase binding effector protein 2 (RABEP2) is a primed substrate for Glycogen Synthase kinase-3 (GSK3).

by
Lisa Logie[1], Lidy Van Aalten[1], Axel Knebel[2], Thomas Force[3], C. James Hastie[4], Hilary MacLauchlan[4], David G Campbell[2], Robert Gourlay[2], Alan Prescott[5], Jane Davidson[6], Will Fuller[1] and Calum Sutherland*[1].

*corresponding author
Division of Molecular and Clinical Medicine, Ninewells Medical School, University of Dundee, Dundee DD1 9SY
email: c.d.sutherland@dundee.ac.uk

1Division of Molecular and Clinical Medicine, University of Dundee
2 MRC Protein Phosphorylation and Ubiquitylation Unit, University of Dundee
3 Department of Cardiovascular Medicine, Vanderbilt University, TN, USA
4 Division of Signal Transduction and Therapy, University of Dundee
5 College of Life Sciences, University of Dundee, Dundee
6 Division of Cancer, University of Dundee

Original Images for the cropped gels and blots used in main article

Gel 1-Fig 1E autorad

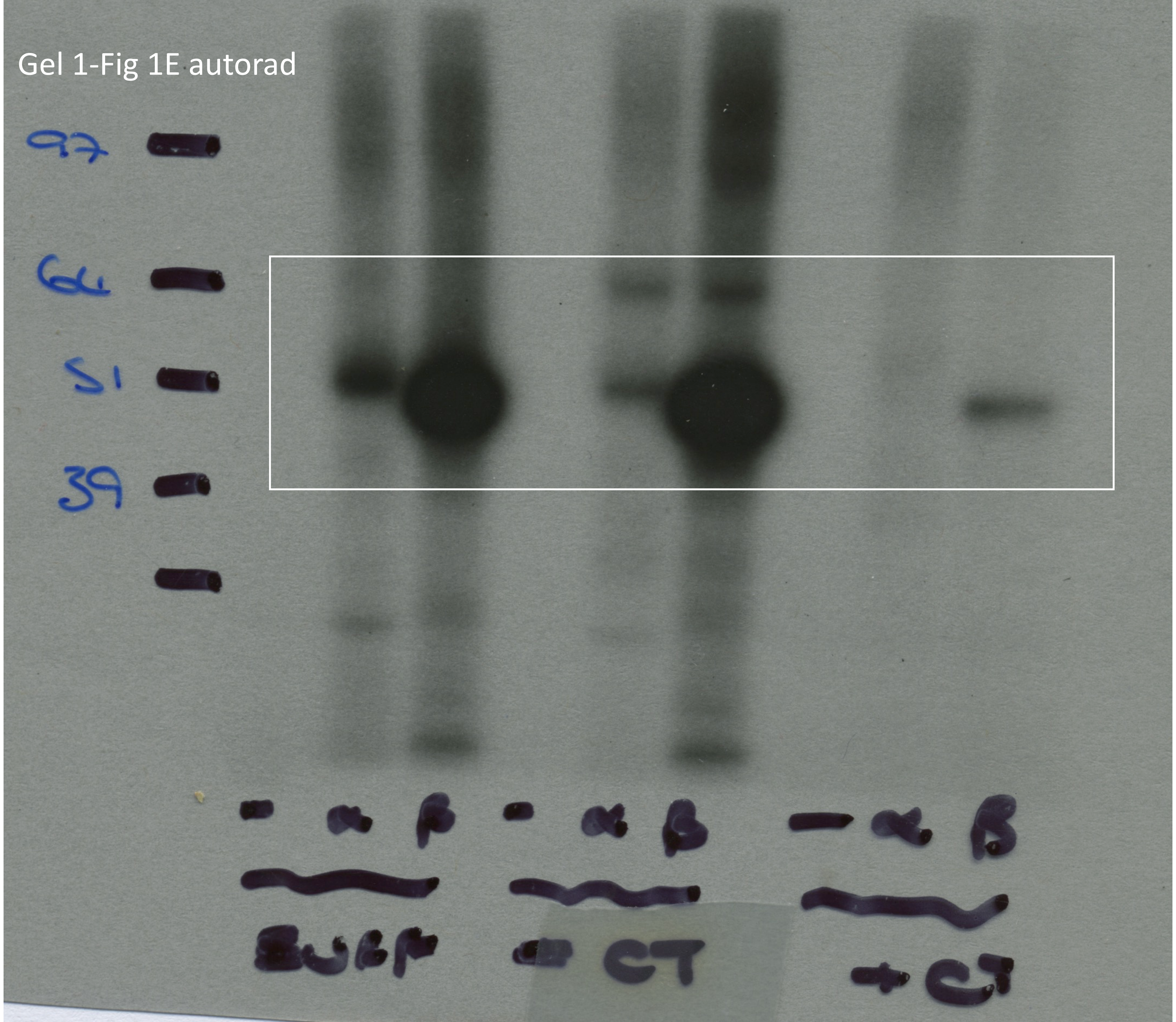

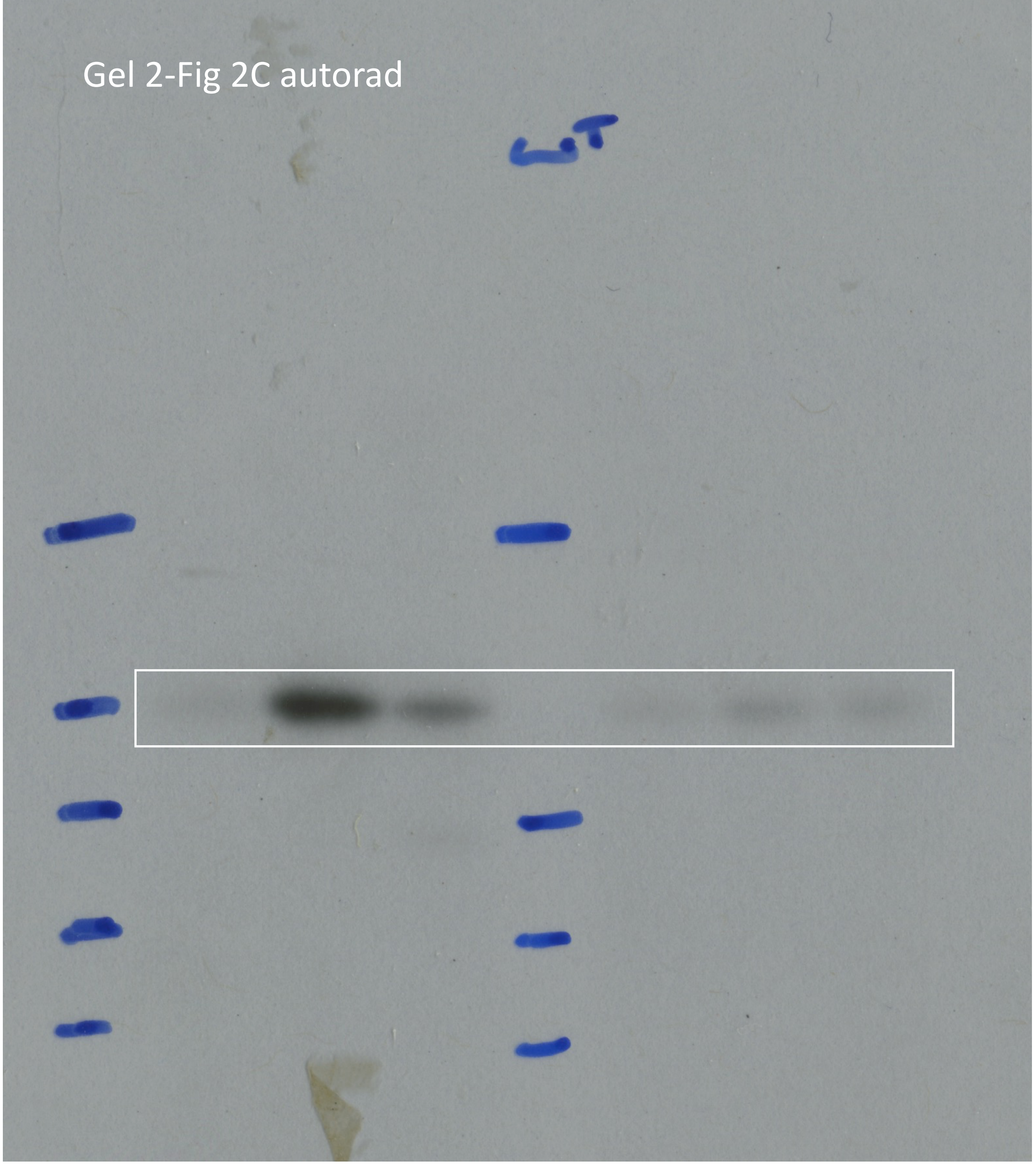
Gel 2-Fig 2C autorad
WT

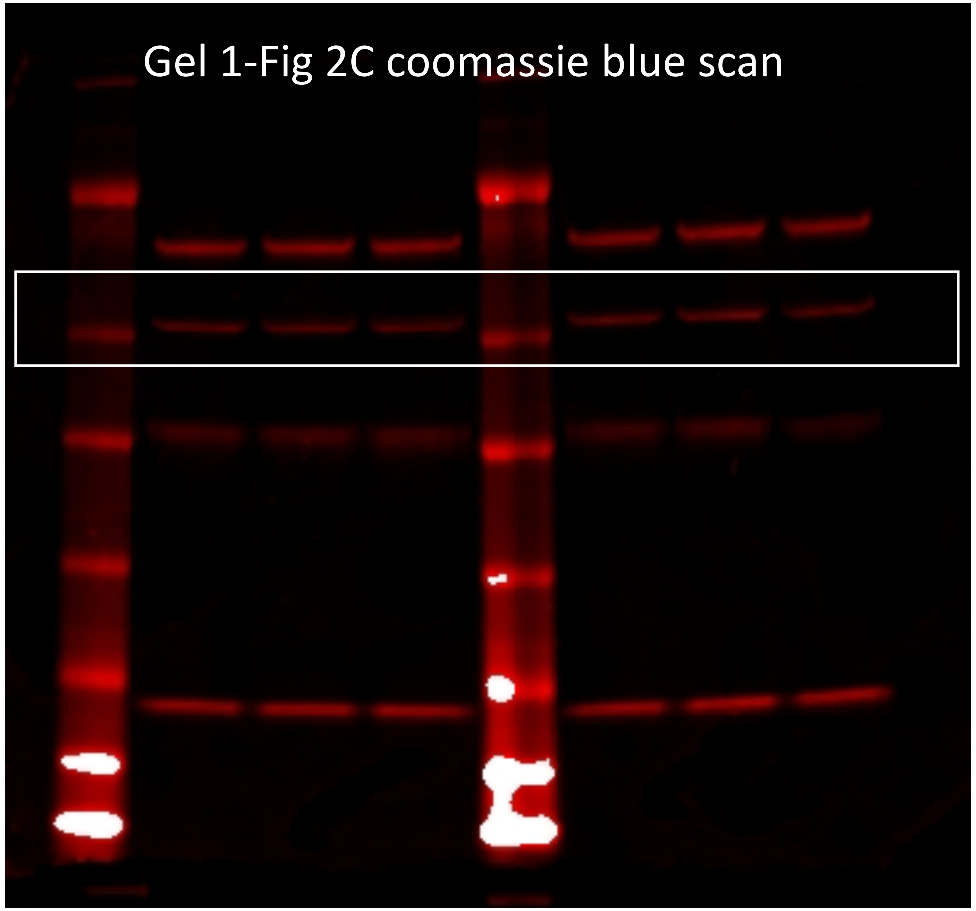
Gel 1-Fig 2C coomassie blue scan

Gel 3-new Fig 2D western developed by Odyssey
Merged image of the p200 and total rabep2 shown, while membrane generated from transfer of same gel was cut at 55kDa and lower half exposed to GSK3 primary antibody separately, prior to scanning.
It is shown
below right.

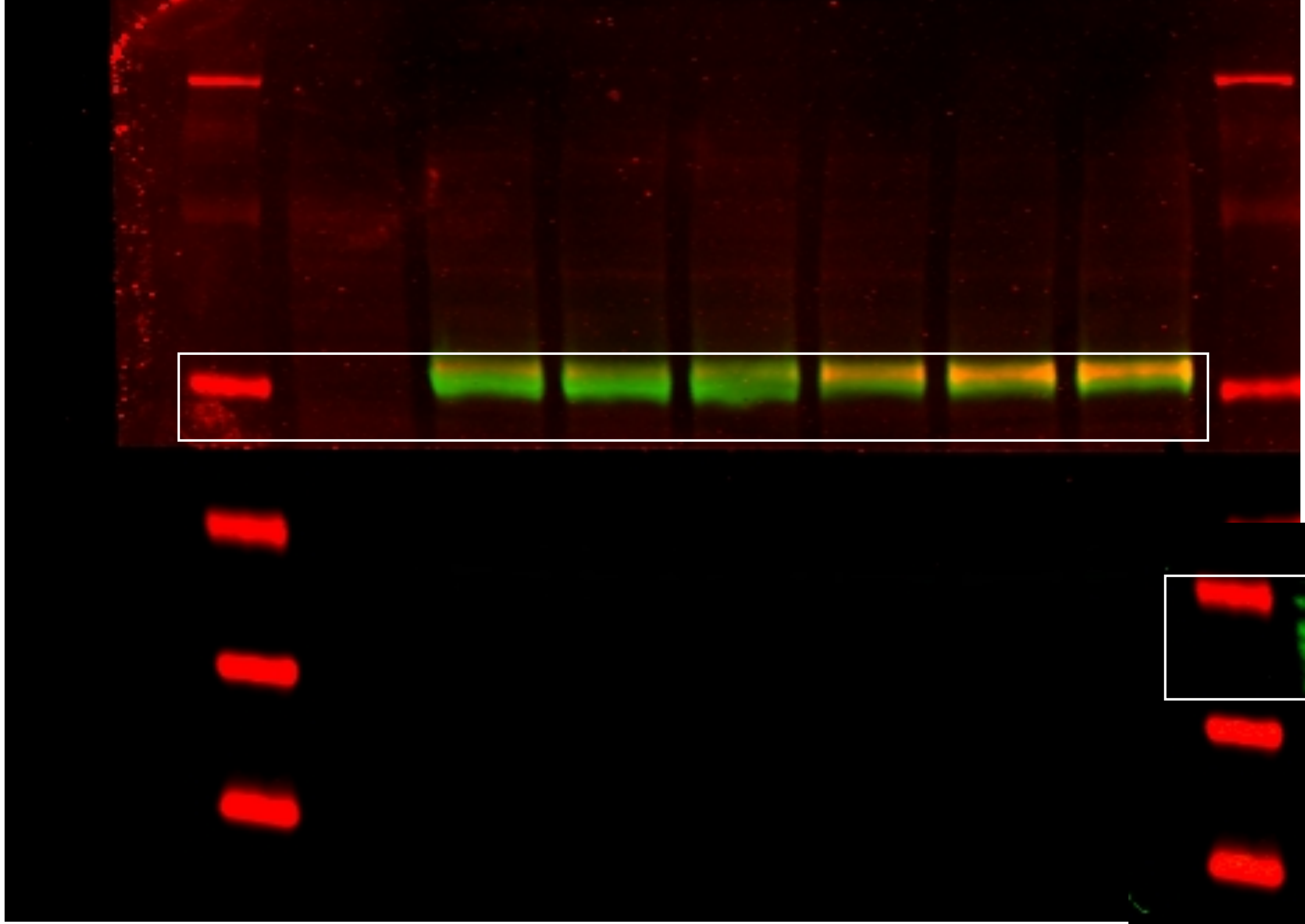

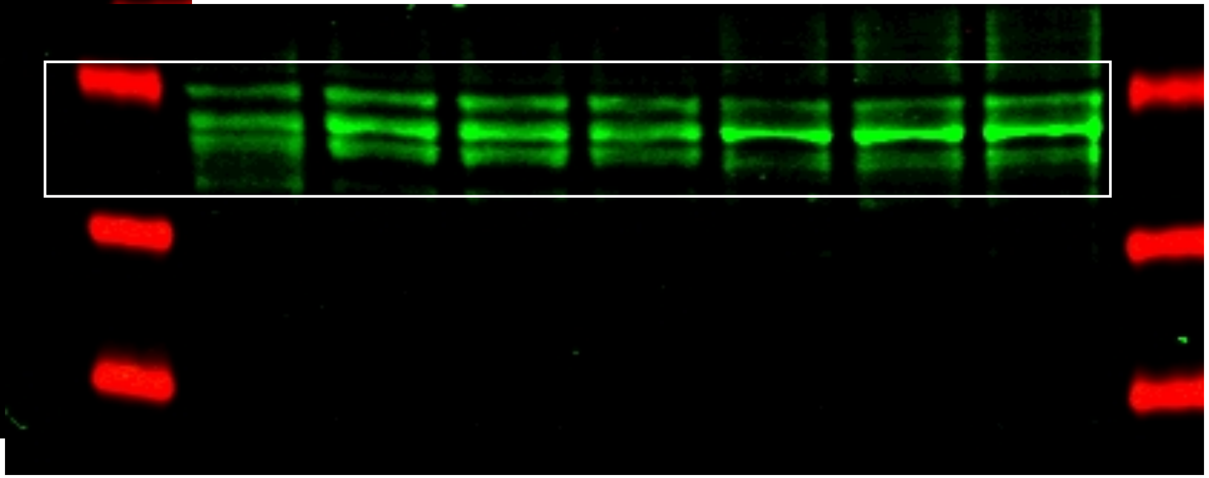

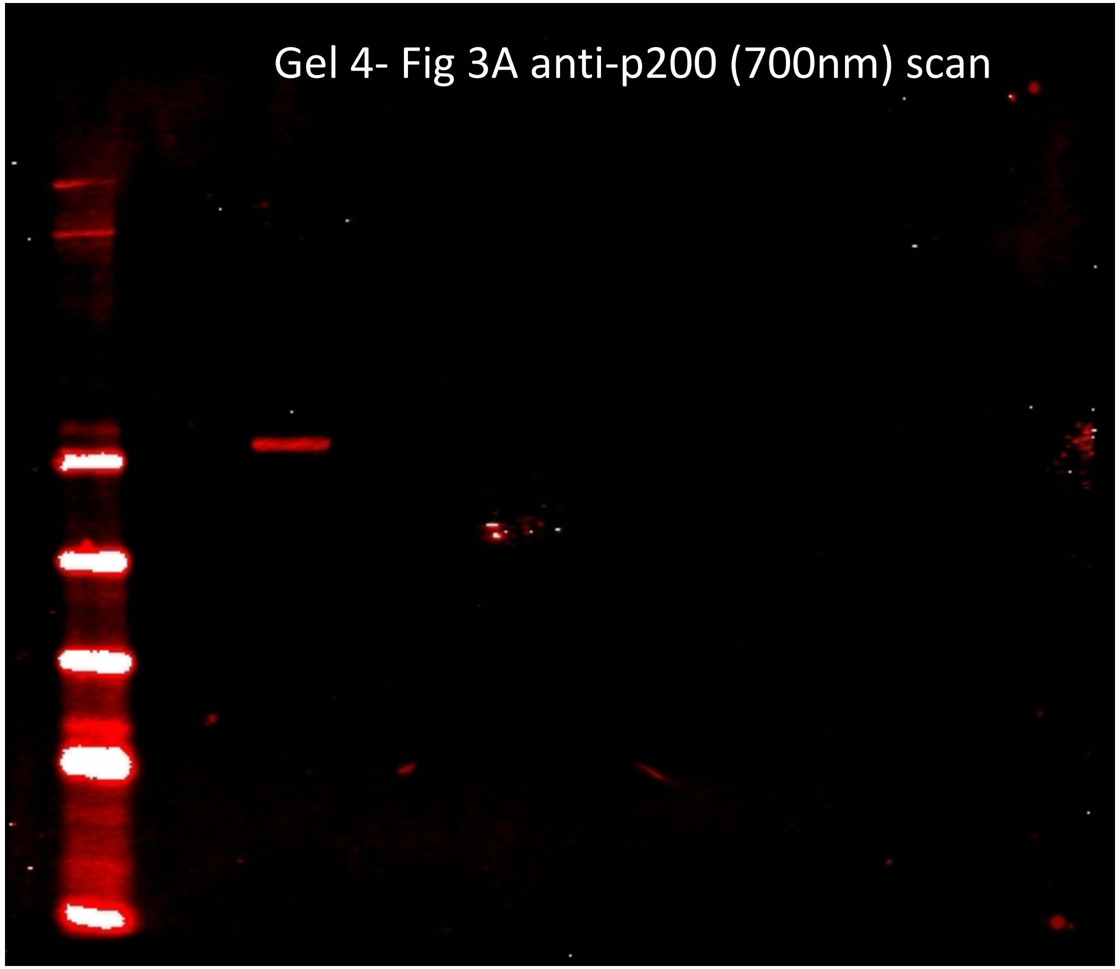
Gel 4- Fig 3A anti-p200 (700nm) scan

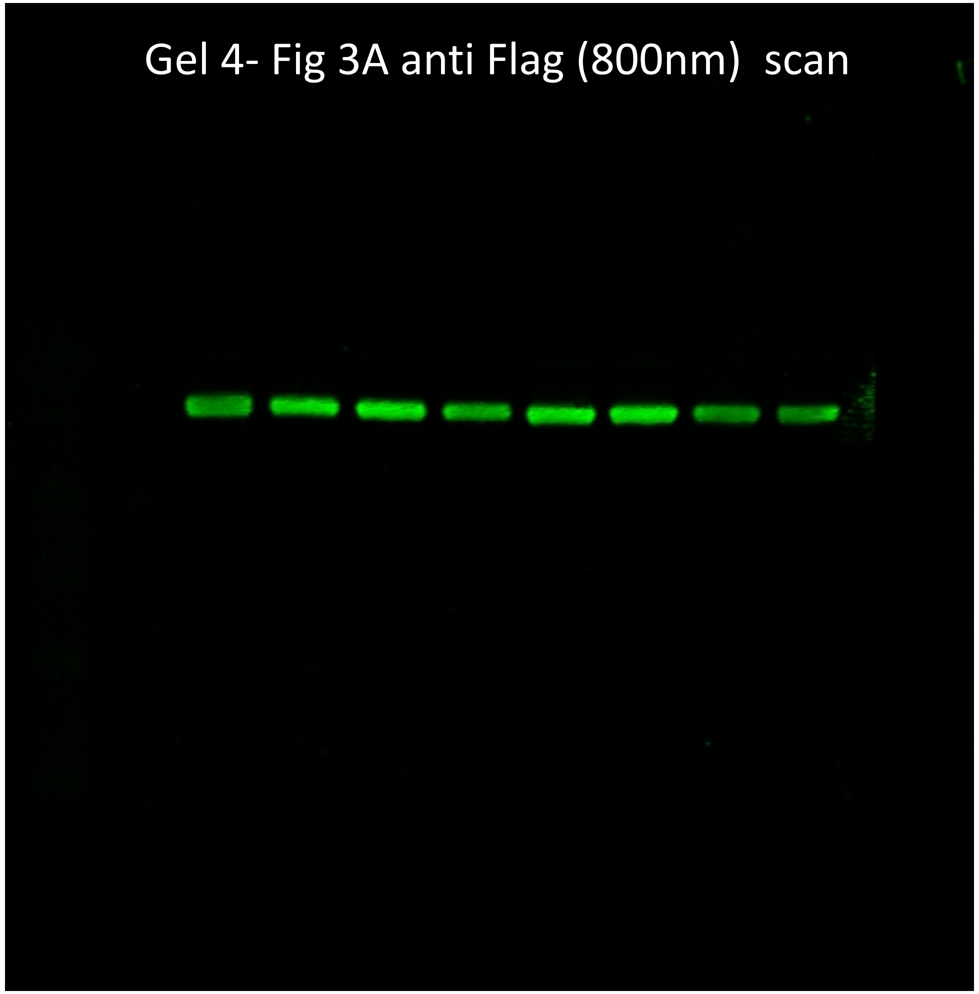
Gel 4- Fig 3A anti Flag (800nm) scan

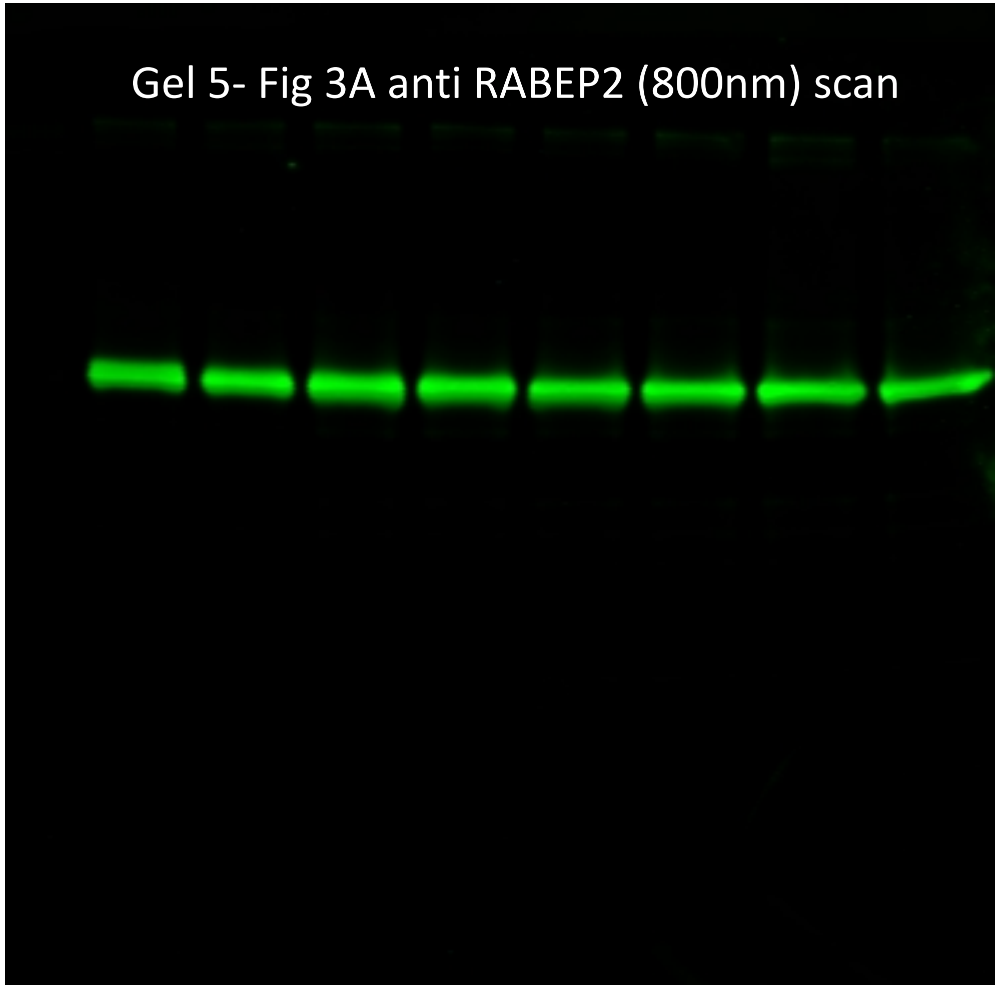
Gel 5- Fig 3A anti RABEP2 (800nm) scan

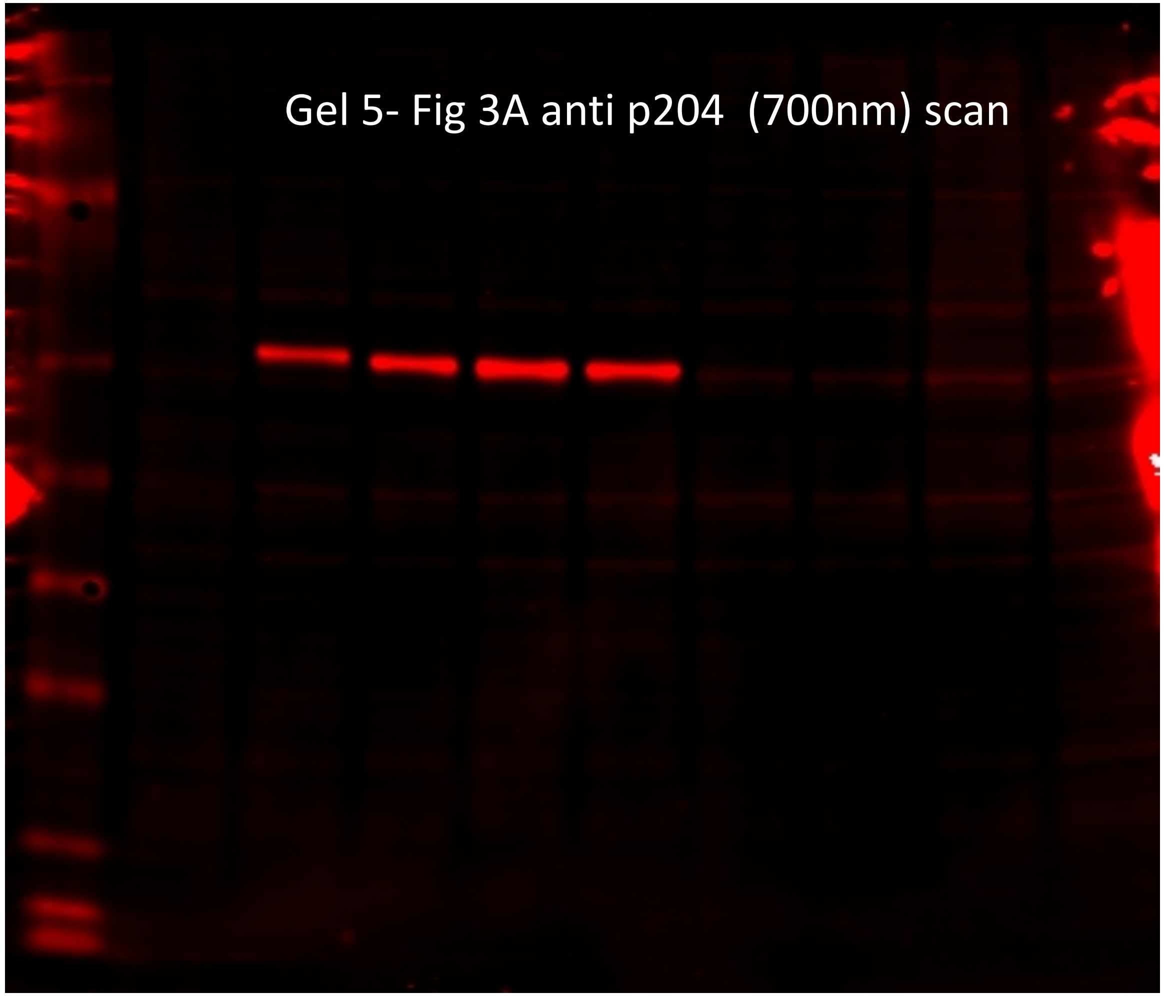
Gel 5- Fig 3A anti p204 (700nm) scan

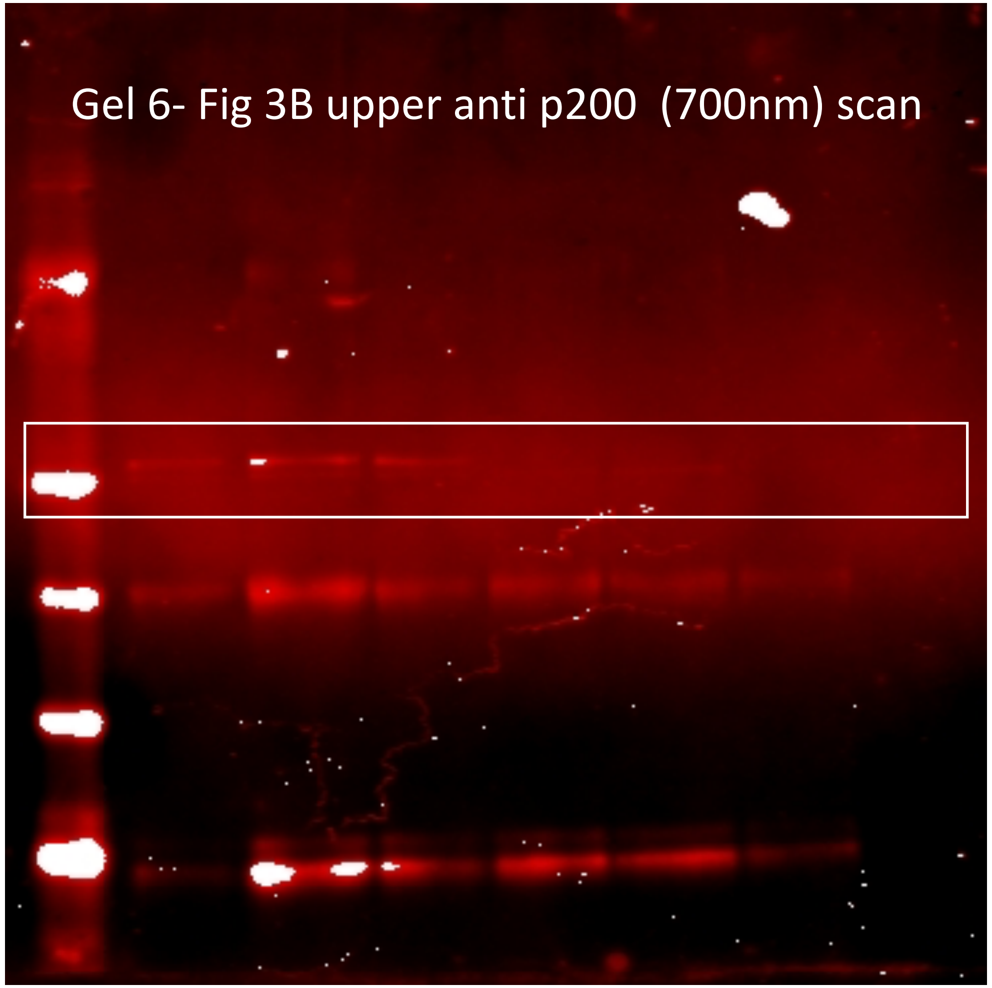
Gel 6- Fig 3B upper anti p200 (700nm) scan

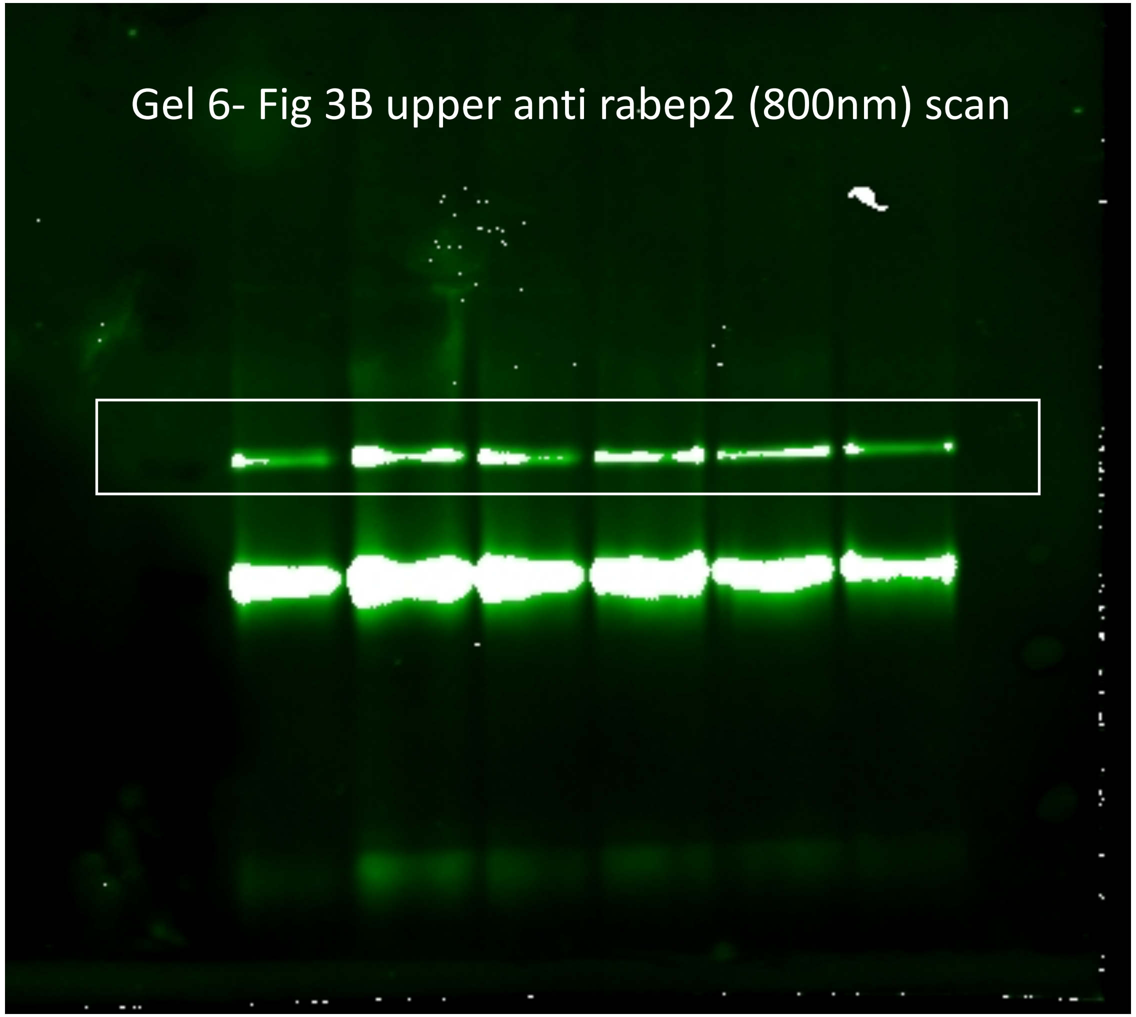
Gel 6- Fig 3B upper anti rabep2 (800nm) scan

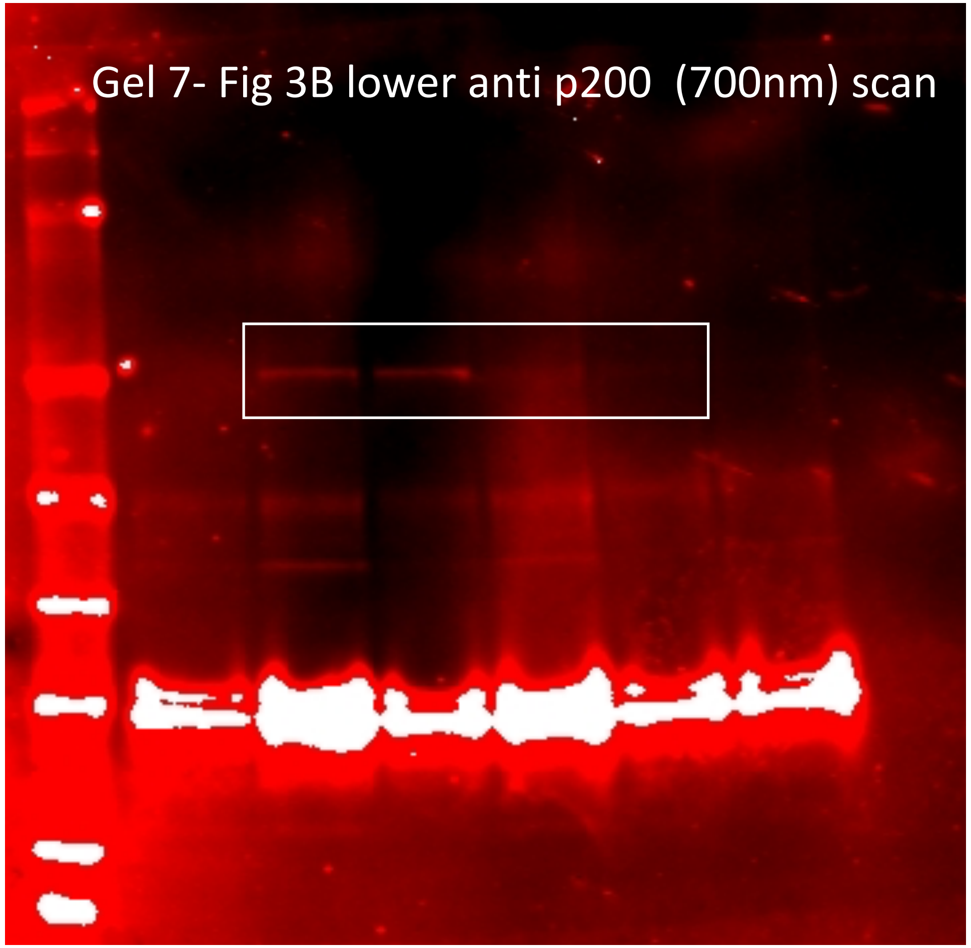
Gel 7- Fig 3B lower anti p200 (700nm) scan

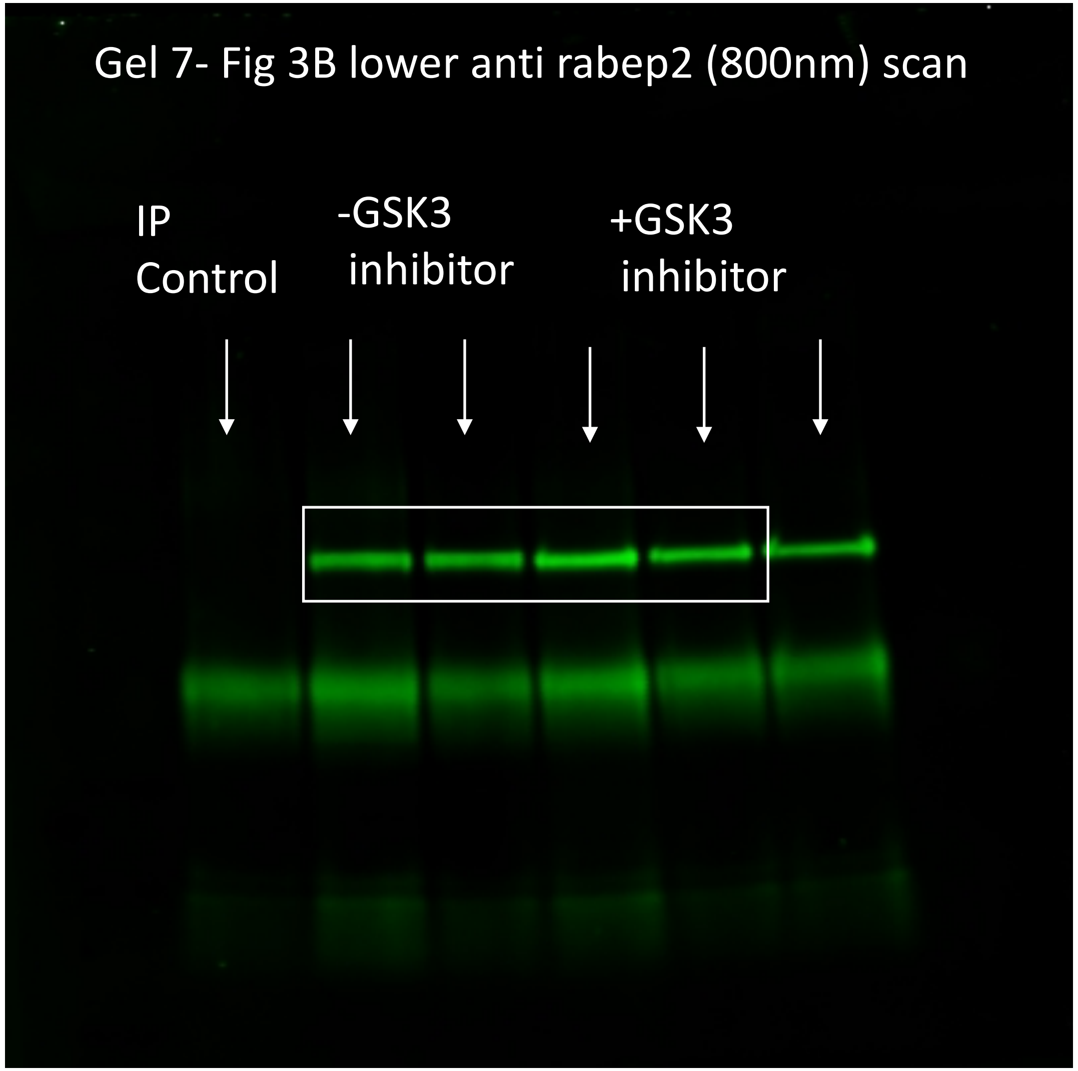
Gel 7- Fig 3B lower anti rabep2 (800nm) scan
IP Control
-GSK3 inhibitor
+GSK3 inhibitor

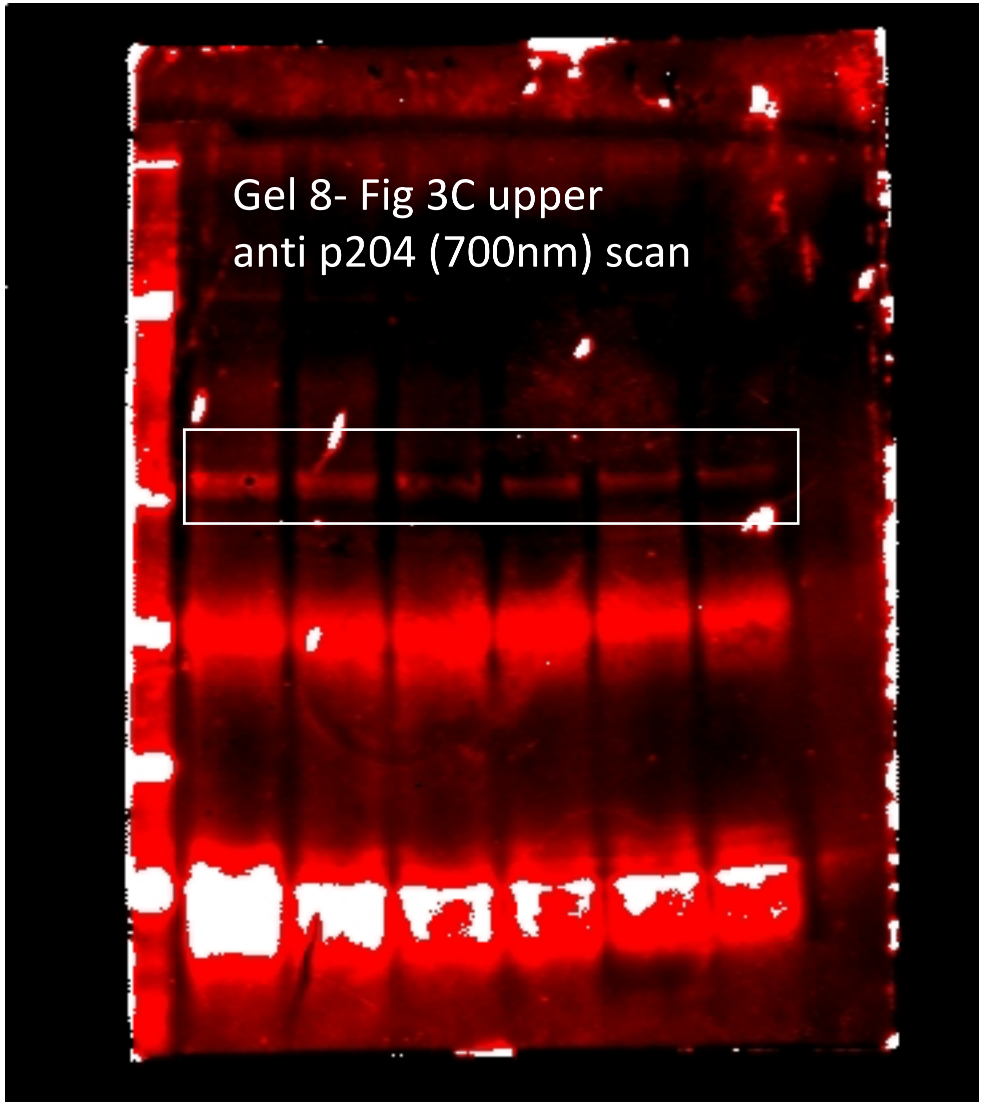

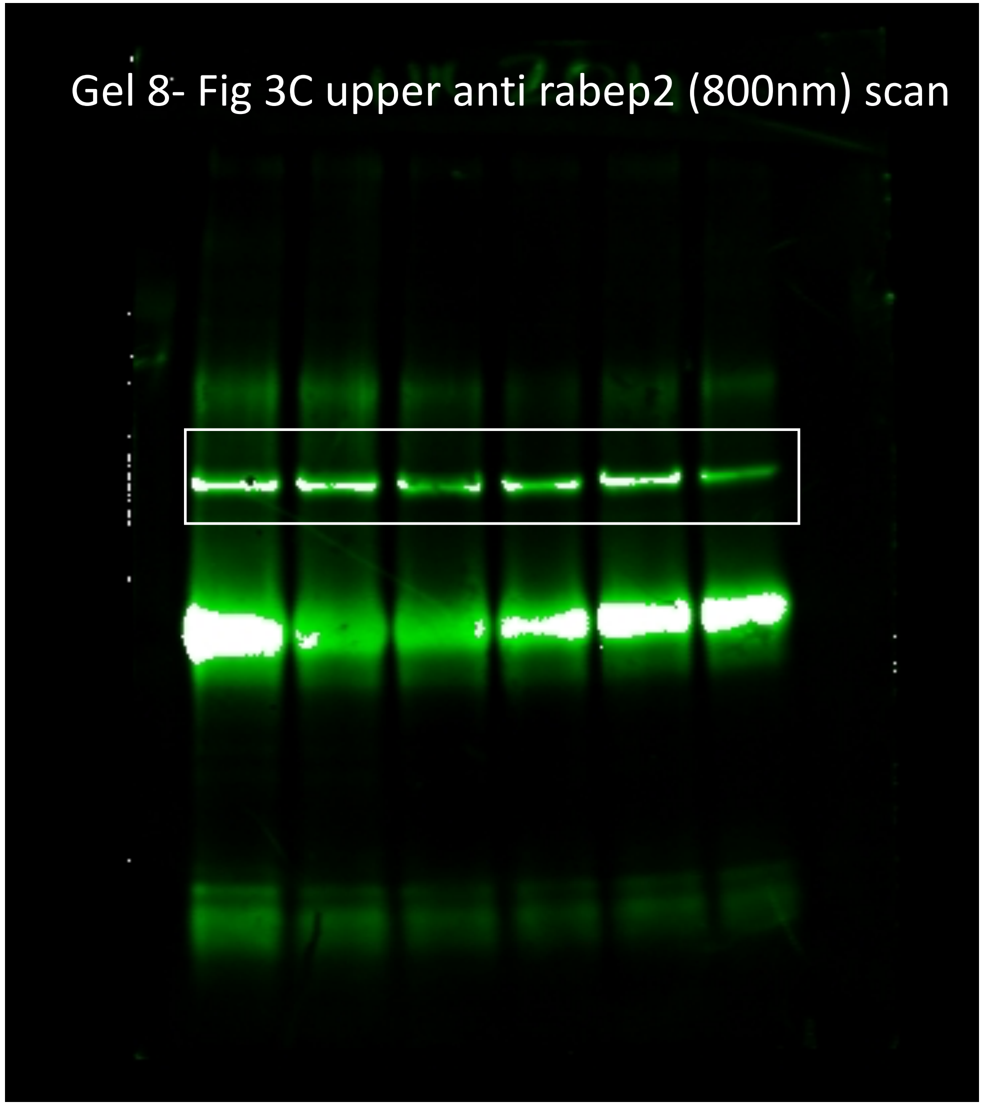

Rescanned 60-70 kDa region at lower intensity laser

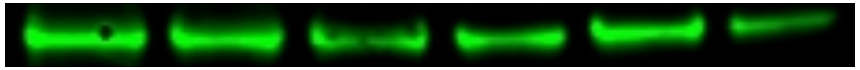

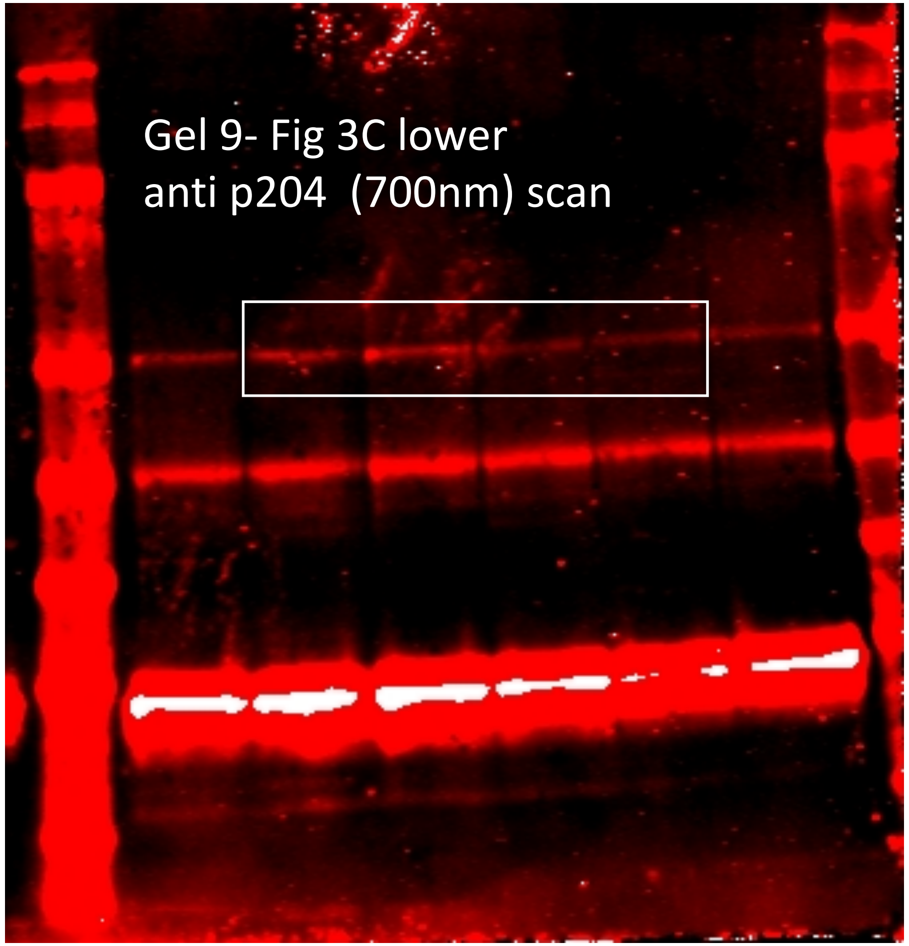

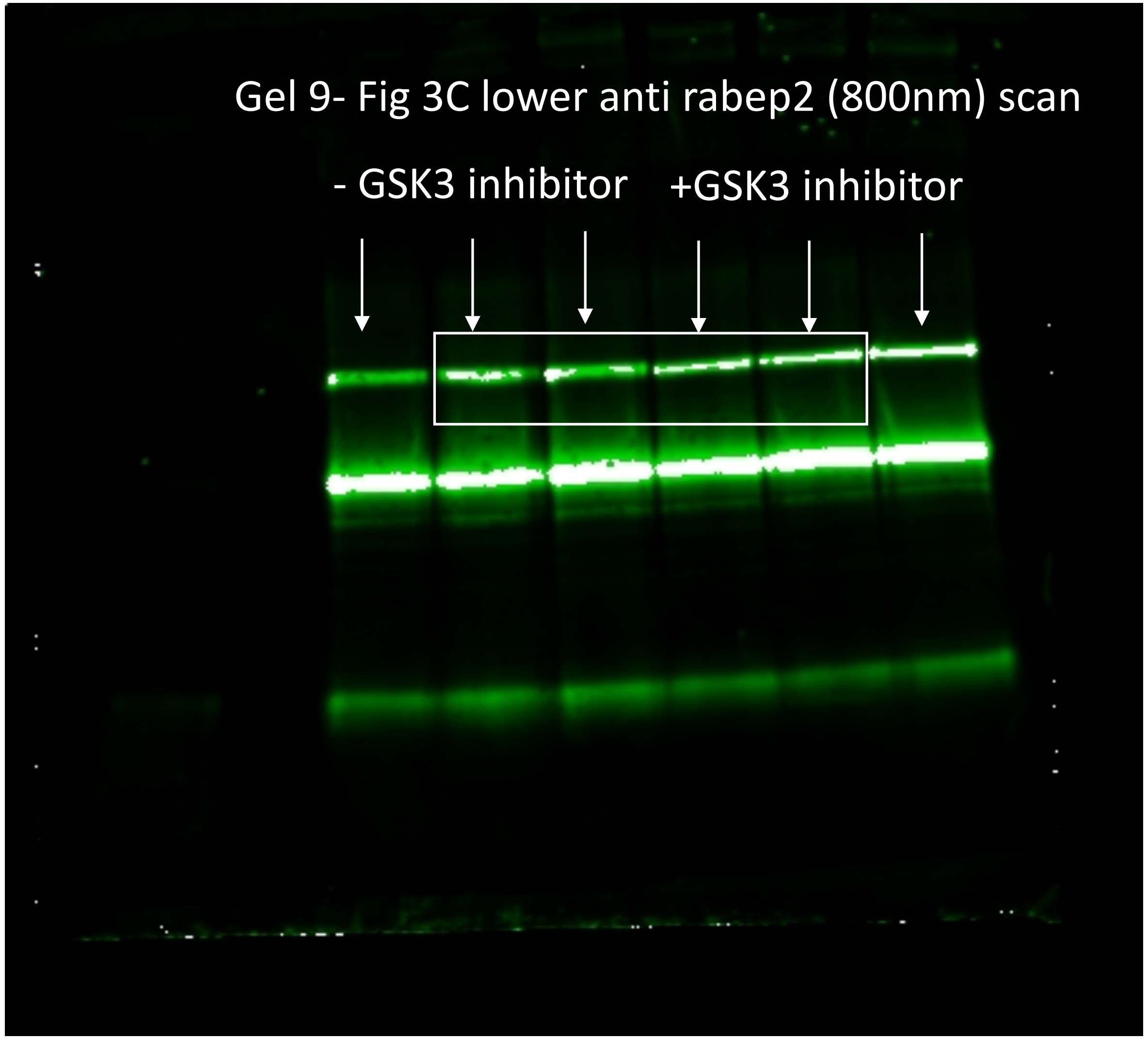

Rescanned 60-70 kDa region at lower intensity laser

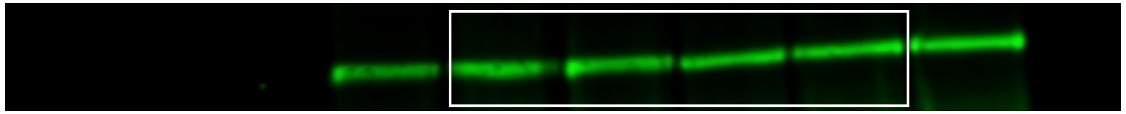

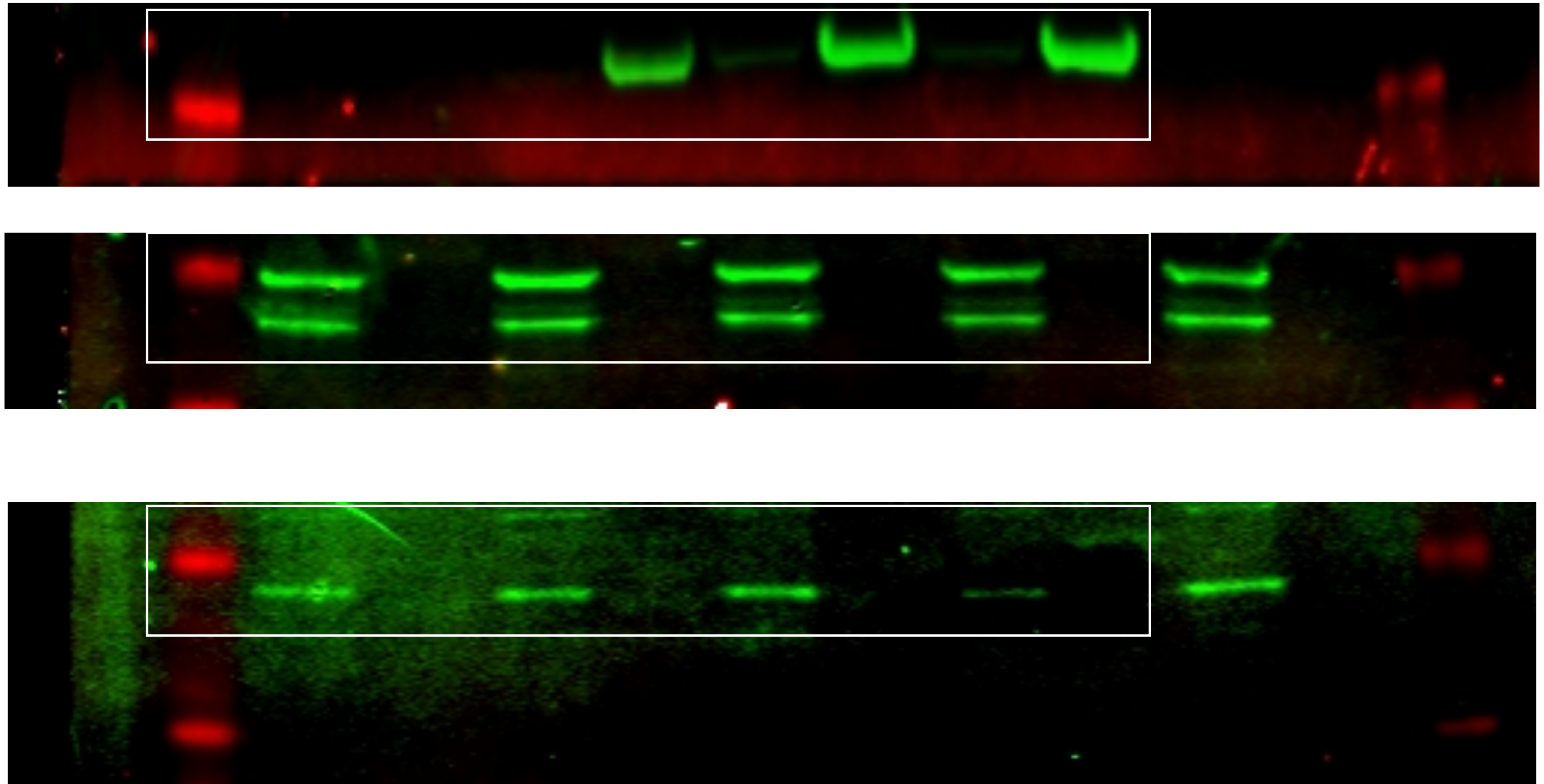

Gel 10- Fig 4A- same gel transferred to membrane and cut into 3 by molecular mass
Upper probed with total RABEP2, middle with GSK3 and lower with Rab5.

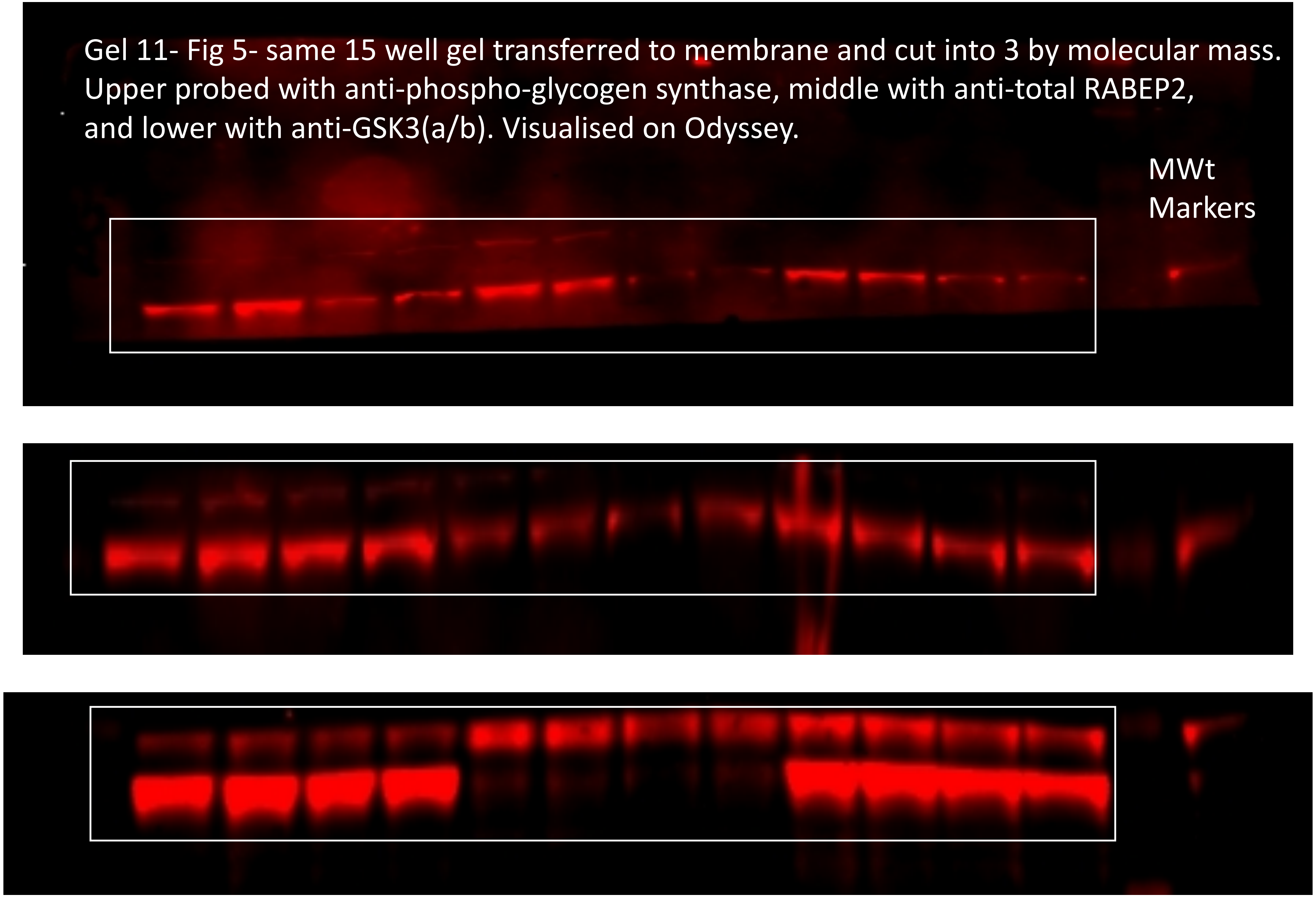
Gel 11- Fig 5- same 15 well gel transferred to membrane and cut into 3 by molecular mass.
Upper probed with anti-phospho-glycogen synthase, middle with anti-total RABEP2,
and lower with anti-GSK3(a/b). Visualised on Odyssey.
MWt
Markers
